# Systematic evaluation of tumor microenvironment and construction of a machine learning model to predict prognosis and immunotherapy efficacy in triple-negative breast cancer based on data mining and sequencing validation

**DOI:** 10.3389/fphar.2022.995555

**Published:** 2022-09-26

**Authors:** Qiheng Gou, Zijian Liu, Yuxin Xie, Yulan Deng, Ji Ma, Jiangping Li, Hong Zheng

**Affiliations:** ^1^ Department of Medical Oncology of Cancer Center, West China Hospital, Sichuan University, Chengdu, China; ^2^ Laboratory of Molecular Diagnosis of Cancer, Clinical Research Center for Breast, West China Hospital, Sichuan University, Chengdu, Sichuan, China; ^3^ Department of Medical Oncology, West China Hospital, Sichuan University, Chengdu, China; ^4^ Institute of Thoracic Oncology and Department of Thoracic Surgery, West China Hospital, Sichuan University, Chengdu, China

**Keywords:** triple-negative breast cancer, tumor microenvironment, machine learning model, prognosis, immunotherapy efficacy

## Abstract

**Background:** The role of the tumor microenvironment (TME) in predicting prognosis and therapeutic efficacy has been demonstrated. Nonetheless, no systematic studies have focused on TME patterns or their function in the effectiveness of immunotherapy in triple-negative breast cancer.

**Methods:** We comprehensively estimated the TME infiltration patterns of 491 TNBC patients from four independent cohorts, and three cohorts that received immunotherapy were used for validation. The TME subtypes were comprehensively evaluated based on immune cell infiltration levels in TNBC, and the TRG score was identified and systematically correlated with representative tumor characteristics. We sequenced 80 TNBC samples as an external validation cohort to make our conclusions more convincing.

**Results:** Two TME subtypes were identified and were highly correlated with immune cell infiltration levels and immune-related pathways. More representative TME-related gene (TRG) scores calculated by machine learning could reflect the fundamental characteristics of TME subtypes and predict the efficacy of immunotherapy and the prognosis of TNBC patients. A low TRG score, characterized by activation of immunity and ferroptosis, indicated an activated TME phenotype and better prognosis. A low TRG score showed a better response to immunotherapy in TNBC by TIDE (Tumor Immune Dysfunction and Exclusion) analysis and sensitivity to multiple drugs in GDSC (Genomics of Drug Sensitivity in Cancer) analysis and a significant therapeutic advantage in patients in the three immunotherapy cohorts.

**Conclusion:** TME subtypes played an essential role in assessing the diversity and complexity of the TME in TNBC. The TRG score could be used to evaluate the TME of an individual tumor to enhance our understanding of the TME and guide more effective immunotherapy strategies.

## Introduction

Worldwide, breast cancer, accounting for approximately 30% of cancers in women (Siegel et al., 2020), can be divided into three subtypes based on estrogen receptor (ER), progesterone receptor (PR), and HER2 status: hormone receptor-positive, HER2-positive, and triple-negative breast cancer (TNBC) (Denkert et al., 2017). TNBC, characterized by a lack of ER, PR, and HER2 expression, accounts for approximately 15%–20% of all breast cancers (Adams et al., 2019a; Marra et al., 2019; Michel et al., 2020). Higher local recurrence and distant metastasis rates than other breast cancer subtypes are outstanding characteristics of TNBC, resulting in the worst overall survival (OS). Approximately 30% of TNBC patients suffer recurrence within 5 years of diagnosis (Lin et al., 2012); therefore, selecting populations suitable for different treatments for TNBC patients is crucial.

However, previous studies have emphasized the significance of cell–cell interactions and upregulated signaling pathways in regulating the tumor microenvironment (TME) (Quail and Joyce, 2013; Su et al., 2018), suggesting that whole sample intercellular relationships are more vital than transcriptional variations of tumor cells (Kalluri, 2016; Mantovani et al., 2017). The TME conditions at the baseline level could reflect the immunotherapy efficacy and chemotherapy response rate (Rosenberg et al., 2016), and various TME cells, such as cytotoxic T cells, tumor-associated macrophages (TAMs), dendritic cells (DCs), and cancer-associated fibroblasts (CAFs), were correlated with therapeutic benefits in various tumors, including breast cancer and melanoma and urothelial cancer (Lee et al., 2014; Nishino et al., 2017; Mariathasan et al., 2018). Understanding the TME instead of cancer cells seems to be a promising method for determining the heterogeneity in breast cancer, and various cells in the TME should be completely described and analyzed (Cagan and Meyer, 2017; Mejia-Pedroza et al., 2018). Previous studies reported that TNBC was characterized by more abundant immune cell infiltration and higher levels of immune checkpoint inhibitor expression than other breast cancer subtypes (Mittendorf et al., 2014; Denkert et al., 2018; Loi et al., 2019). Some studies have shown that high levels of lymphocytic infiltration, such as CD8^+^ T and CD4^+^ T cells, are consistently correlated with a more favorable prognosis in TNBC (Savas et al., 2016; Jang et al., 2018; Gao et al., 2020).

Although it is challenging to treat TNBC patients and they are usually treated with standard chemotherapy and PARP inhibitors (Robson et al., 2017; Telli et al., 2018), several clinical trials have reported that immunotherapy might improve the survival of TNBC patients. For instance, the IMpassion130 trial implied that atezolizumab was beneficial in previously untreated metastatic TNBC (Schmid et al., 2018). The Keynote355 trial reported that pembrolizumab benefited the PDL-1-positive TNBC population in terms of PFS (Cortes et al., 2020). Although these findings reinforce the perspective that immunotherapy seems more appropriate for TNBC, considerable research is urgently needed to identify benefit groups from this therapeutic strategy.

A previous study depicted a vast TME landscape of gastric cancer and helped to provide new strategies for interpreting responses to immunotherapies (Zeng et al., 2019). Considering the lack of rigorous studies on the TME subtype in TNBC, with the emergence of more analytical techniques, two TME-related subtypes were identified by clustering of immune cell infiltration levels. Based on TME-related genes and the machine learning method PCA algorithm, a TME-related gene (TRG) scoring system for TNBC patients was constructed and validated in several public datasets and validation cohorts sequenced by ourselves. There were several studies based on this dimensionality reduction method, such as an m6A-related score from our previous study (Liu et al., 2021), a “writer” score model for colorectal cancer (Chen et al., 2021), and a mast cell-based signature in lung cancer (Bao et al., 2020). All of these studies constructed a scoring system based on differentially expressed genes among several identified subtypes. Meanwhile, our TRG score in TNBC was also highly associated with the activation of related pathways, the cancer stemness index, and drug sensitivity.

Most importantly, the TRG score was further employed to predict the immunotherapy responses in TIDE analysis, revealing that we could determine the benefit populations of TNBC patients who received immunotherapy. Interestingly, a 20-member prognostic signature simplified by the iterative LASSO algorithm could predict the survival probability of TNBC patients and could shrink the TRG score calculation members, which had the same ability as the TRG score. Eventually, all of these analysis results were validated in a TNBC cohort with sequencing data and clinical information by ourselves.

## Materials and methods

### Data sources and filtering

The raw data were downloaded from the Gene Expression Omnibus (GEO) (https://www.ncbi.nlm.nih.gov/geo/) and Cancer Genome Atlas (TCGA) databases. Three TNBC datasets [GSE96058 (Brueffer et al., 2018), GSE86166 (Prabhakaran et al., 2017), and GSE103091 (Jezequel et al., 2015)] and two datasets related to immunotherapy [GSE35640 (Ulloa-Montoya et al., 2013) and GSE78220 (Hugo et al., 2016)] in the GEO database were used for analysis. The pan-cancer data involving 17 cancer types in TCGA were downloaded from the UCSC XENA database (https://xenabrowser.net/datapages/) (Goldman et al., 2020). We extracted TNBC data from all TCGA datasets for the principal analysis, and other tumors were used for validation. Moreover, the profiles of the IMvigor210 cohort were obtained according to official guidelines (http://research-pub.Gene.com/imvigor210corebiologies) (Mariathasan et al., 2018). All of the information of the public datasets is summarized in Supplementary Table S1.

### Tissue sample collection and high-throughput sequencing

In addition, we used a cohort constructed by the West China Hospital breast cancer specialist research team as an external validation cohort, including 80TNBC biopsies, and this experiment was approved by the Ethics Committee of West China Hospital. Total RNA was extracted and purified following the manufacturer’s protocol. After synthesizing first- and second-strand cDNA using random hexamer primers, DNA polymerase I and RNase H, the library fragments were purified with an AMPure XP system (Beckman Coulter, Beverly, MA, United States) as described in the NEBNext UltraTM Directional RNA Library following the manufacturer’s recommendations. The libraries were then sequenced on the Illumina HiSeq X ten platform (Novogene Bioinformatic Technology Co., Ltd., China) following a 150 bp paired-end read protocol. Eventually, the raw sequencing data from this study have been deposited in the Genome Sequence Archive (GSA) in BIG Data Center (https://bigd.big.ac.cn/) (Zhang et al., 2021a), Beijing Institute of Genomics (BIG), Chinese Academy of Sciences, under the accession number HRA002256.

### Assessment of immune cell infiltration levels

Single-sample gene set enrichment analysis (ssGSEA) is a well-known method to derive the absolute enrichment scores of previously experimentally validated gene signatures conducted by the R package “GSVA,” a nonparametric and unsupervised method commonly employed to estimate the variations in the pathway and biological process activity of a single sample (Li et al., 2017). Here, we preferred to use ssGSEA to assess the relative abundance of immune cell infiltration levels in a single sample. Two validated immune cell signatures published, labeled immune cell signatures 1 and 2 in this study, were used in this research, containing 24 (Bindea et al., 2013) and 23 (Charoentong et al., 2017) types of immune cells, respectively. The markers of these two signatures are listed in Supplementary Tables S2, S3. To further validate the results from ssGSEA, the CIBERSORT algorithm (Newman et al., 2015), which is a deconvolution algorithm, was employed to infer cell-type proportions with bulk tumor sequence data. Moreover, the third method, called Estimation of Stromal and Immune cells in malignant tumors using Expression data (ESTIMATE) (Yoshihara et al., 2013), was also used to infer the fraction of stromal and immune cells in tumor samples.

### Functional enrichment analysis

Using ssGSEA described previously, GSVA (Hanzelmann et al., 2013) was used to assess pathway activation levels in a single sample with the gene set “c5.all.v6.2. symbols” downloaded from the MSigDB database in GSEA website (Mootha et al., 2003) and another published pathway gene set summarized in Supplementary Table S4 (Mariathasan et al., 2018). GO and Kyoto Encyclopedia of Genes and Genomes (KEGG) analyses were conducted using the R package and the online website Database for Annotation, Visualization, and Integrated Discovery (DAVID) (david.ncifcrf.gov) (Dennis et al., 2003).

### Unsupervised clustering and differentially expressed gene analysis

Unsupervised clustering analysis was used to classify patients based on the immune cell infiltration levels with the ConsensuClusterPlus package (Wilkerson and Hayes, 2010). Differentially expressed gene (DEGs) analysis was conducted by the “limma” R package, with the criterion of adjusted *p* value < 0.05. The differentially expressed mRNAs were visualized by the “pheatmap” package.

### Calculation of the ferroptosis index and mRNA-based stemness index

A total of 113 ferroptosis regulators were extracted from the online website FerrDb (http://www.zhounan.org/ferrdb/), and the specific information of these genes is shown in Supplementary Table S5. To describe the ferroptosis level, the ferroptosis index (FPI) was established based on the expression data of genes in ferroptosis, including positive and negative components. The enrichment score (ES) was calculated using ssGSEA, and the FPI to roughly assess ferroptosis trends was calculated as follows:

FPI = ES (positive) − ES (negative) (Liu et al., 2020)

To assess the stemness of cancer cells, a one-class logistic regression algorithm known as mRNA-based stemness index (mRNAsi) was used to calculate the stemness index for each sample under the direction of the workflow available on a previously established database (https://bioinformaticsfmrp.github.io/) (Malta et al., 2018).

### Therapeutic response prediction

The chemotherapeutic response for TNBC was predicted according to the data involved in the Genomics of Drug Sensitivity in Cancer (GDSC) with the “pRRophetic” package (Geeleher et al., 2014). The Tumor Immune Dysfunction and Exclusion (TIDE) database (http://tide.dfci.harvard.edu/) was employed to predict the immunotherapy response of TNBC (Jiang et al., 2018), and the default cutoff value was 0.

### Calculation of tumor microenvironment-related gene score

The overlapping DEGs among the four TNBC datasets were regarded as TME gene signatures. Principal component analysis (PCA) was used to calculate the TRG score to quantize the TME patterns in TNBC. We summed PC1 and PC2 of genes i by PCA as described before by us (Liu et al., 2021). The TRG score was calculated as follows:

TRG score = Σ (PC1i + PC2i)

### Prognostic signature construction and survival analysis

Logistic least absolute shrinkage and selection operator (LASSO) regression analysis can construct a prognostic signature to minimize the risk of overfitting (Simon et al., 2011). However, LASSO relies heavily on seeds when it allows. Iteration LASSO was independent of the seed once the roots, the optimal lambda, and the resulting feature were changed (Sveen et al., 2012). The features retained at high frequency can be considered the most influential factors. Genes included under consensus were generated by iteration of LASSO, and AUC further selected the minimum combination of genes associated with survival. The formula of patients’ risk scores was established:

Risk score = Σ (each gene’s expression × corresponding coefficient).

Receiver operating characteristic (ROC) curves and survival curves with the Kaplan–Meier method were used to judge the prediction efficiency of the signature. The best cutoff value of genes in survival analysis was searched by the “survminer” R package. The signature genes obtained from iterated LASSO analysis were used for nomogram construction using logistic and Cox regression analyses. Calibration curves were used to assess the predictive accuracy of the nomogram.

### Statistical analysis

Correlation coefficients and *p* values were calculated by Spearman correlation analysis among several defined groups. Wilcoxon tests were used to compare differences between the two groups. The asterisks represent the statistical *p* values (*p < 0.05, **p < 0.01, and ***p < 0.001) in the panels.

## Results

### Identification of tumor microenvironment subtypes

**FIGURE 1:**
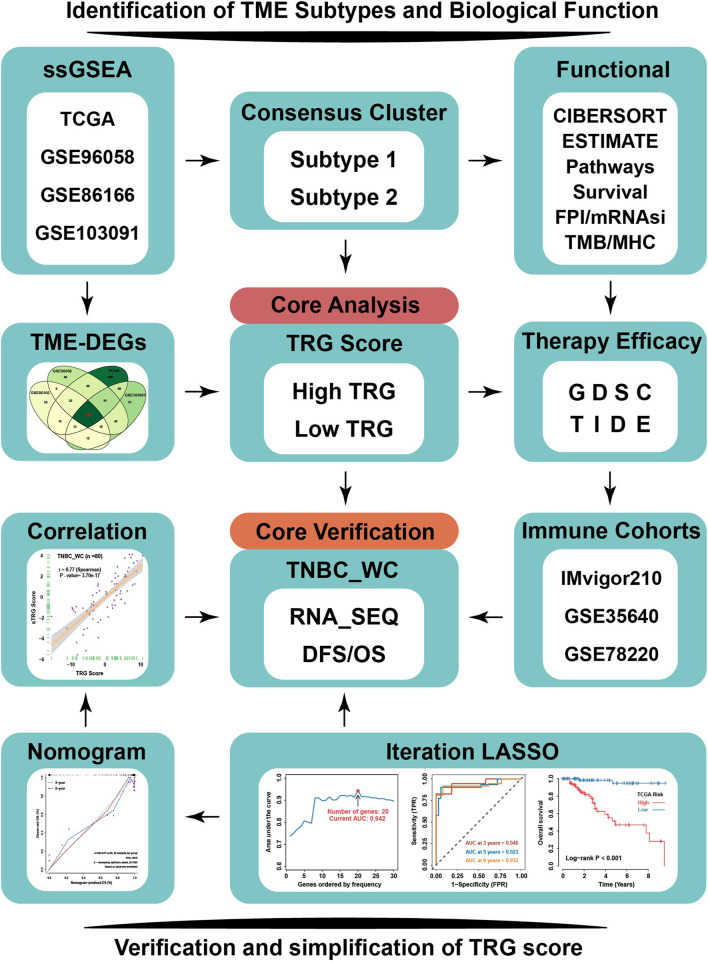
Flowchart of our research. TNBC patients from four independent TNBC cohorts (TCGA, GSE96058, GSE86166, and GSE103091) were used to conduct an unsupervised clustering analysis based on relative immune cell infiltration levels. Two TME subtypes were identified and systematically correlated with representative tumor characteristics. TRG score based on TME-related DEGs calculated by machine learning was employed to reflect TME subtypes’ attributes. TRG score could predict response to immunotherapy and sensitivity to multiple drugs in TNBC by TIDE and GDSC analysis. TNBC_WC samples as an external validation cohort to verify the effectiveness of TRG score, and based on iteration LASSO analysis, simplified TRG scores involving 20-member prognostic signature were established for clinical use to predict the survival probability of TNBC. TME-DEGs, tumor microenvironment-related differentially expressed genes; FPI, ferroptosis index; mRNAsi, mRNA-based stemness index; GDSC, Genomics of Drug Sensitivity in Cancer; TIDE, Tumor Immune Dysfunction and Exclusion; TRG, TME-related genes; and TNBC_WC, triple-negative breast cancer cohort in West China hospital.

**FIGURE 2:**
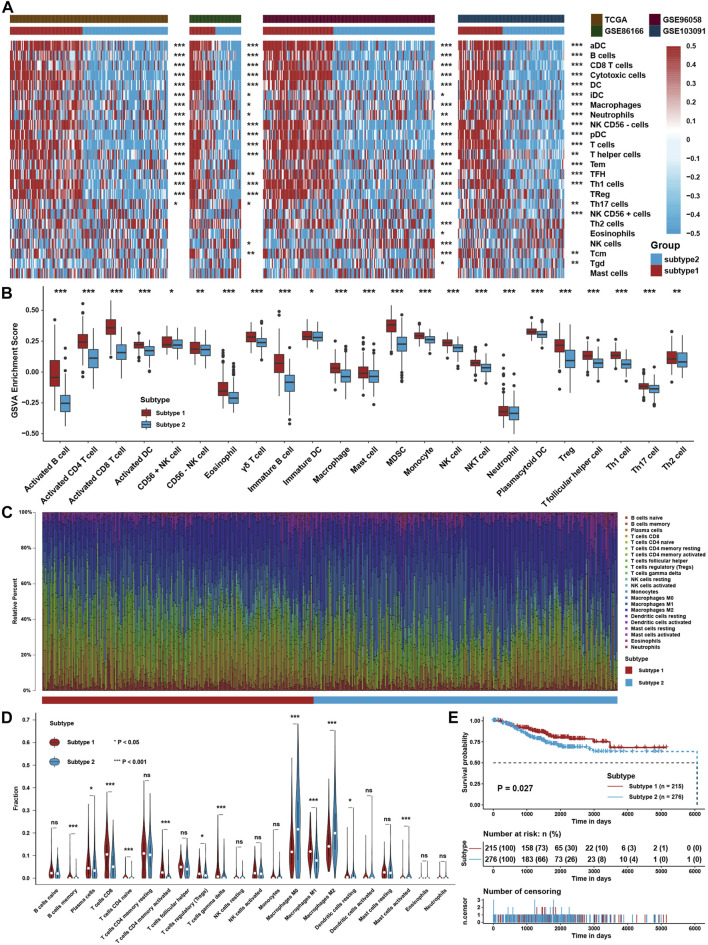
Identification of TME Subtypes. **(A)** Clustering heatmap of immune cell infiltration levels calculated with immune cell signature 1 in four TNBC cohorts. **(B)** Immune cell infiltration levels were calculated with immune cell signature 2 in the TNBC cohort between the two TME subtypes. **(C)** CIBERSORT algorithm assessed the relative percentage of different immune cell types in a single sample in the whole TNBC cohort. **(D)** Differences in immune cell type relative percentages assessed by the CIBERSORT algorithm in a single sample in the whole TNBC cohort. **(E)** Survival analysis for TME subtypes in the whole TNBC cohort. The asterisks represent the statistical *p* value (*p < 0.05; **p < 0.01; ***p < 0.001).

The flowchart of this study is depicted in Figure 1. To explore the tumor microenvironment patterns in four independent TNBC cohorts, consensus cluster analysis was used to classify patients with TME conditions (Supplementary Figures S1A–D). By integrating the clustering results of each dataset, two distinct TME subtypes were eventually identified using unsupervised clustering in each cohort, labeled as subtypes 1 and 2 (Figure 2A). Here, we used immune cell signature 1 to perform cluster analysis. At the same time, we found that the infiltration of the levels of immune cells was significantly different using immune cell signature 2 in all four cohorts (Figure 2B). Among them, subtype 1 was enriched with immune cells compared with subtype 2, meaning that subtype 1 was an immune-activating subtype with higher immune cell infiltration levels, the same as the conception of a “hot” tumor. By the CIBERSORT algorithm, we found that some antitumor immune cells, such as CD8+ T cells, activated CD4+ T cells, and M1-like macrophages, were elevated in subtype 1. In contrast, tumor-associated immune cells, such as M2-like macrophages, were more elevated in subtype 2 (Figures 2C,D). Given these differences in the TME for these two subtypes, survival analysis showed that the overall survival of subtype 1 in the four cohorts was better than that of subtype 2 (Figure 2E).

### Biological function analysis between tumor microenvironment subtypes

**FIGURE 3:**
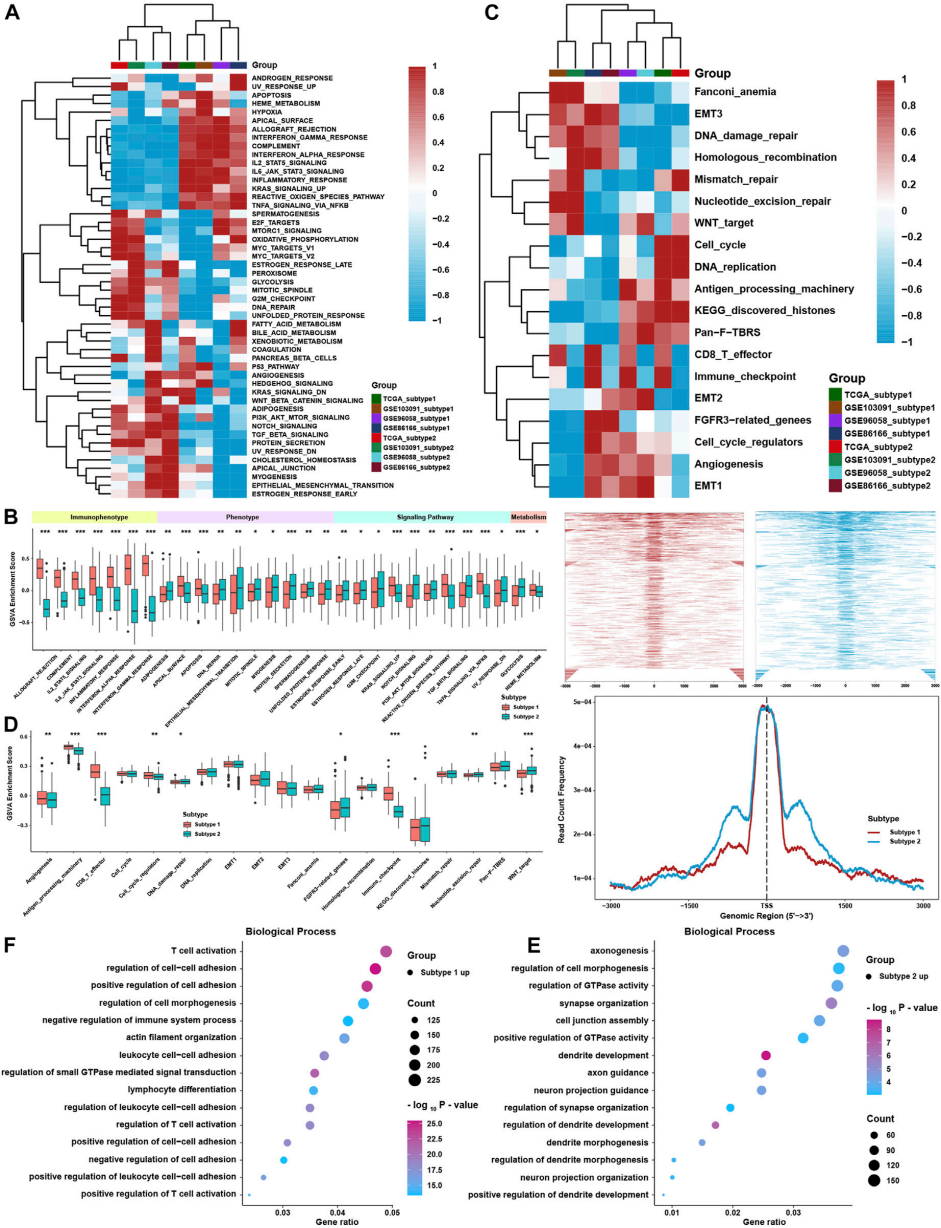
Biological function analysis between TME subtypes. **(A)** Heatmap of the GSVA enrichment score in curated pathways in four TNBC cohorts. **(B)** Differences in GSVA enrichment scores in curated pathways in the whole TNBC cohort. **(C)** Heatmap of curated pathways calculated with another pathway signature in four TNBC cohorts. **(D)** Differences in curated pathways were calculated with another pathway signature in the whole TNBC cohort. **(E)** Differentially expressed peaks were identified between the two TME subtypes in TCGA. **(F)** GO analysis of differentially expressed peaks between two TME subtypes in TCGA. The asterisks represent the statistical *p* value (*p < 0.05; **p < 0.01; and ***p < 0.001).

To further investigate the differences between the two TME subtypes, we considered analyzing the biological function variation in the conception of signaling pathways. GSVA showed that all immune-related pathways, such as the IL-2/STAT5, IL-6/STAT3, and interferon response pathways, were enriched in subtype 1, while the TGF-β-, NOTCH-, PI3K/AKT-, and EMT-related pathways were enriched in subtype 2 (Figures 3A,B). ssGSEA with curated signaling pathway signatures showed that the CD8 T effector- and immune checkpoint-related pathways were activated in subtype 1. In contrast, tumor progression-related pathways such as WNT and EMT were activated in subtype 2 (Figures 3C,D). Based on ATAC-seq data from TCGA, differentially expressed peaks were identified between subtypes 1 and 2 (Figure 3E). GO analysis was processed on these differentially expressed peaks annotated by ChIPseeker, and the results showed that genes correlated with T cell activation had higher chromatin activities in subtype 1. In comparison, genes correlated with the regulation of GTPase and cell morphogenesis possessed higher chromatin activities in subtype 2 (Figure 3F).

**FIGURE 4:**
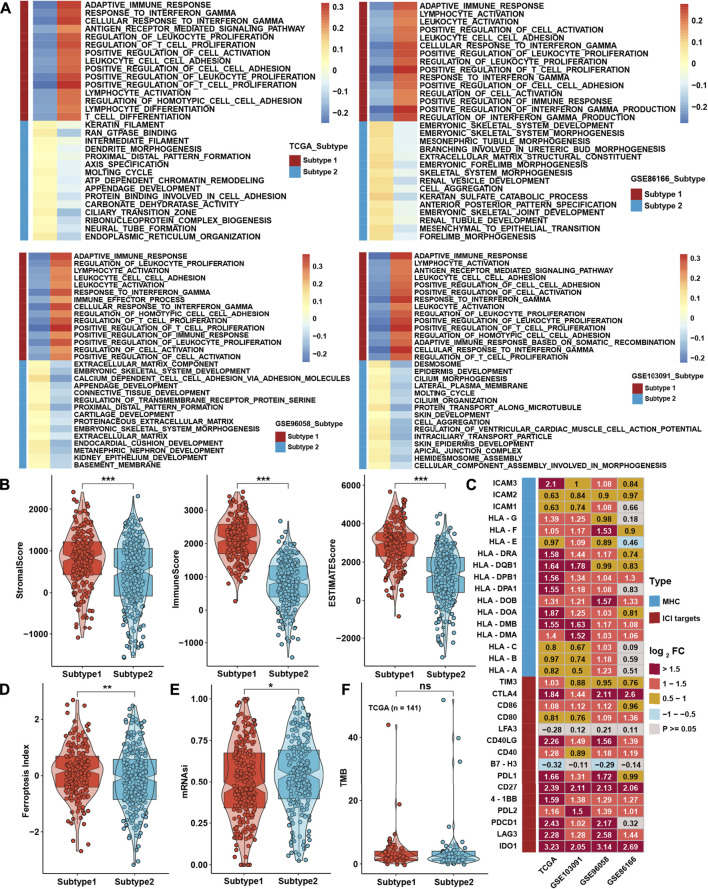
Biological function analysis between TME subtypes. **(A)** GSEA between two TME subtypes in four TNBC cohorts. **(B)** Differences in ESTIMATE analysis results between TME subtypes in the whole TNBC cohort. **(C)** Differential expression analysis of MHC molecules and immune checkpoint inhibitors between TME subtypes in four TNBC cohorts. **(D,E)** Differences in the results for FPI and mRNAsi between TME subtypes in the whole TNBC cohort. **(F)** Differences in TMB between TME subtypes in the TCGA cohort. The asterisks represent the statistical *p* value (*p < 0.05; **p < 0.01; ***p < 0.001).

Moreover, traditional GSEA was also conducted between subtypes in the four cohorts, which was consistent with the abovementioned results (Figure 4A). Using the ESTIMATE method, scores of stromal and immune cells were also higher in subtype 1 (Figure 4B). The expression levels of MHC molecules and immune checkpoint inhibitors (ICIs) are correlated with the activation of the antitumor immune response and the efficacy of immunotherapy. Most MHC molecules and ICIs were significantly different between the two subtypes and were especially higher in subtype 1 (Figure 4C). The abovementioned analysis showed that TME subtype 1 was highly correlated with immune-related phenotypes, while TME subtype 2 was positively associated with tumor progression and metastasis phenotypes. Thus, more comprehensive analyses containing FPI and mRNAsi were employed to analyze the ferroptosis level and the stemness index of single tumor tissue. As we have noticed that the initiation or metastasis of a malignant tumor might be highly correlated with cancer stem cells, we aimed to use mRNAsi to evaluate the differences between two TME subtypes. Moreover, ferroptosis was also a novel and vital phenotype that aroused our interest in further investigating the relationship with immune subtypes; FPI was employed here to satisfy our intention. Eventually, we found that the ferroptosis index (FPI) was higher in subtype 1 than in subtype 2, while the mRNA-based stemness index (mRNAsi) was higher in subtype 2 than in subtype 1 (Figures 4D,E). However, no significant difference was found in tumor mutation burden (TMB) (Figure 4F).

### Generation of tumor microenvironment-related gene score and functional verification

**FIGURE 5:**
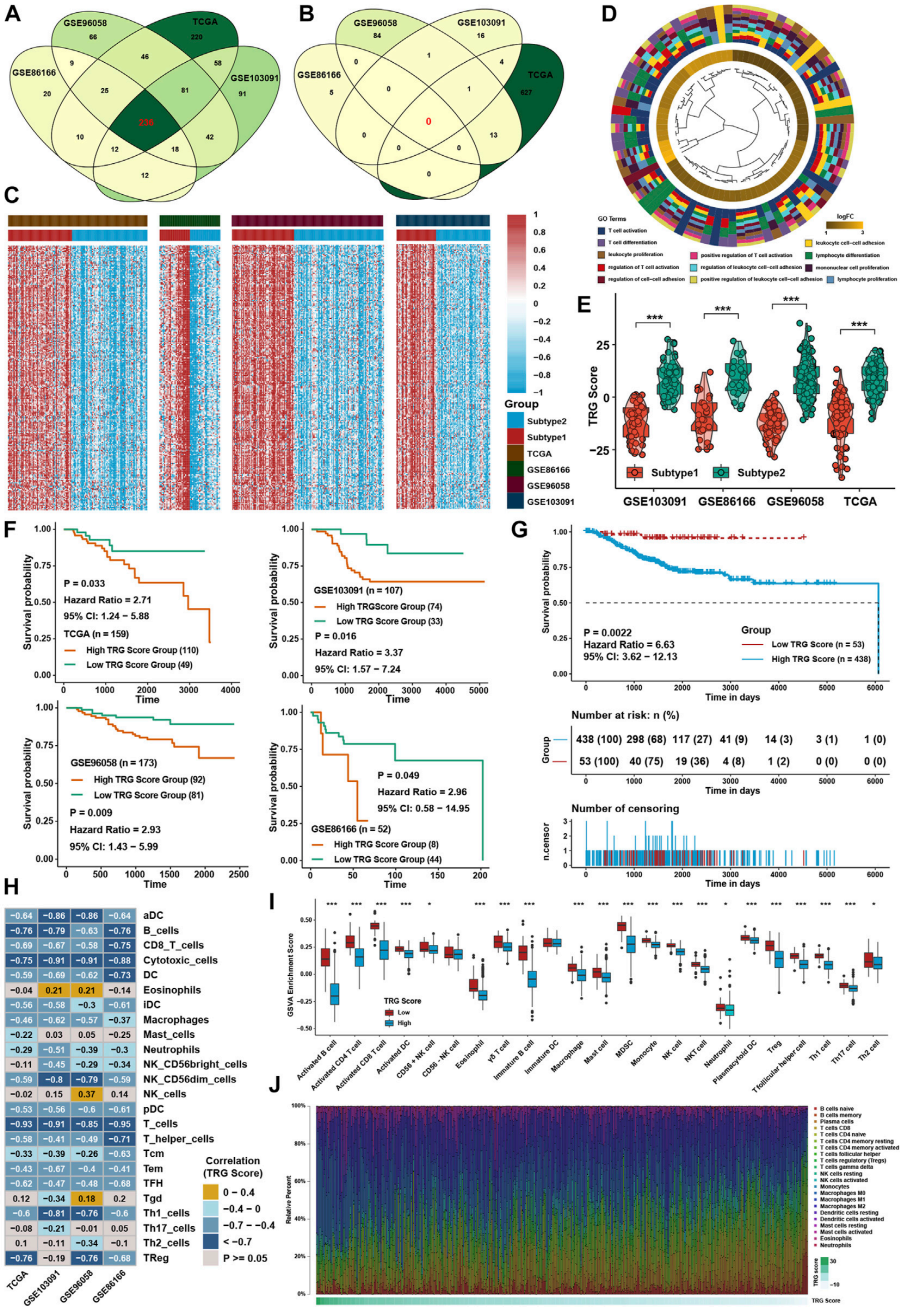
Generation of TRG score and functional verification. **(A)** Intersection of upregulated DEGs between two TME subtypes in four TNBC cohorts. **(B)** Intersection of downregulated DEGs between two TME subtypes in four TNBC cohorts. **(C)** Heatmap of DEGs between two TME subtypes in four TNBC cohorts. **(D)** GO analysis of DEGs intersected between the two TME subtypes. **(E)** Differences in TRG score between TME subtypes in four TNBC cohorts. **(F)** Survival analysis for the TRG score in four TNBC cohorts. **(G)** Survival analysis for the TRG score in the whole TNBC cohort. **(H)** Correlation analysis between the TRG score and immune cell infiltration levels calculated by immune cell signature 1. **(I)** Immune cell infiltration levels calculated with immune cell signature 2 in the whole TNBC cohort between the two TME subtypes. **(J)** Variation tendency of the relative immune cell percentage in a single sample in the whole TNBC cohort. The asterisks represent the statistical *p* value (*p < 0.05; ***p < 0.001).

To further investigate the underlying mechanisms between the two TME subtypes, differentially expressed gene (DEG) analysis was conducted in four TNBC cohorts. Taking the intersection of DEGs in four cohorts (Figures 5A,B), 236 TME-related genes (TRG) were identified between TME subtypes, and all of them were upregulated in subtype 1 (Supplementary Table S6; Figure 5C). GO analysis showed that DEGs were highly enriched in T-cell activation and cell adhesion pathways (Figure 5D). For further analysis, a continuous variable called the TRG score by PCA was generated to quantify the different levels of TME in individual patients. The TRG score could well reflect the differences in TME subtypes in TNBC cohorts, and the TRG score was lower in subtype 1 (Figure 5E). Patients with low TRG score demonstrated a greater survival benefit than patients with high TRG score (Figures 5F,G). ssGSEA calculated with immune cell signature 1 showed that the infiltration levels of most immune cells were highly negatively associated with the TRG score (Figure 5H), and ssGSEA calculated with immune cell signature 2 also verified that most of the immune cells were higher in the low TRG score groups (Figure 5I). CIBERSORT analysis showed that as the TRG score was reduced, the percentage of cytotoxic T cells increased (Figure 5J). GSVA showed that immune-related pathways, such as the IL-2/STAT5, IL-6/STAT3, and interferon response pathways, were negatively correlated with the TRG score.

**FIGURE 6:**
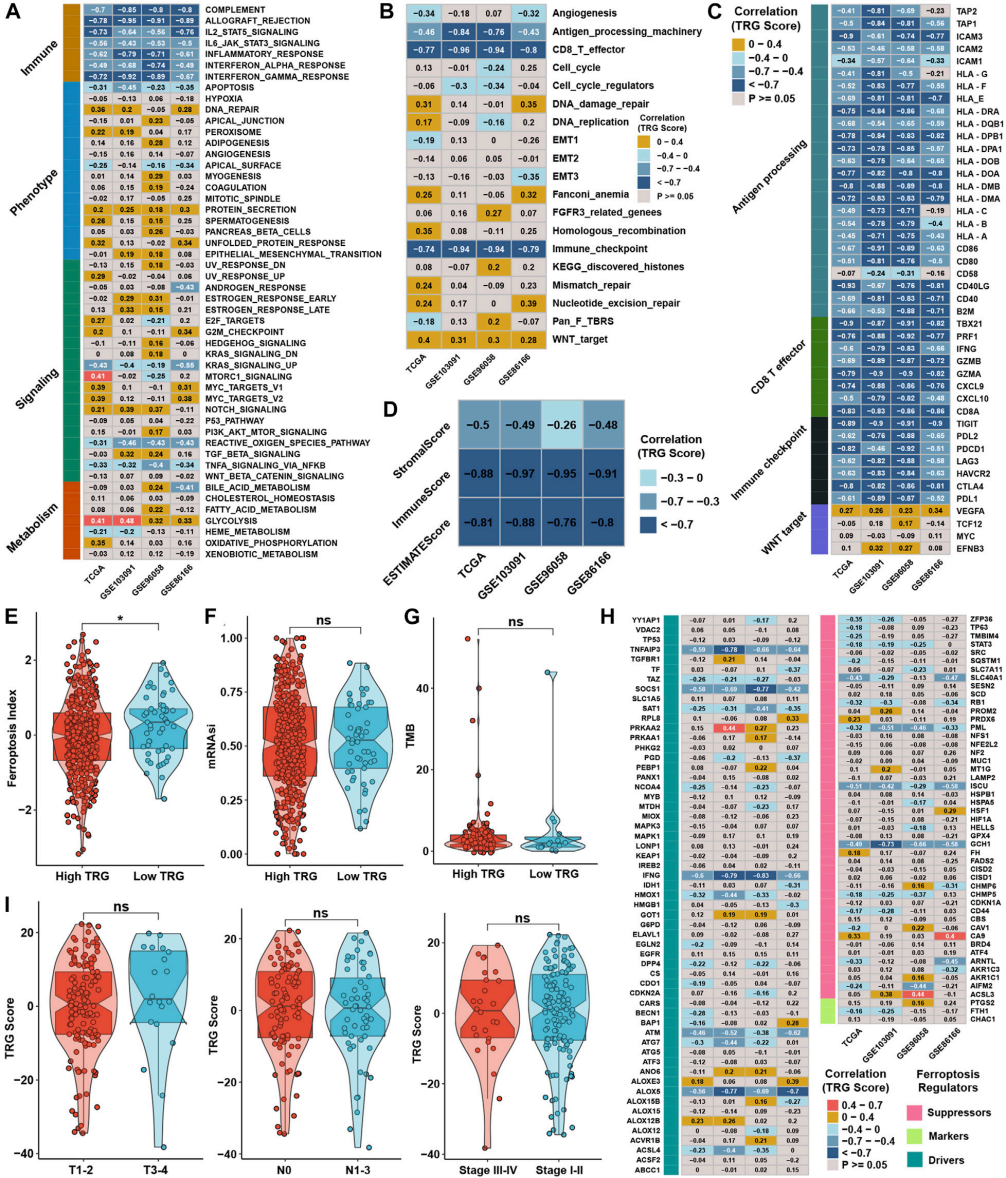
Generation of TRG score and functional verification. **(A)** Correlation analysis between the GSVA score and TRG score in four TNBC cohorts. **(B)** Correlation analysis between the curative pathway enrichment score and TRG score in four TNBC cohorts. **(C)** Correlation analysis between the TRG score and the expression of genes involved in four significant pathways in Supplementary Figure S4C. **(D)** Correlation analysis between the TRG score and the results of ESTIMATE analysis in four TNBC cohorts. **(E,F)** Differences in the results for FPI and mRNAsi between TRG score groups in the whole TNBC cohort. **(G)** Differences in TMB between TRG score groups in the TCGA cohort. **(H)** Correlation analysis between the TRG score and the expression of 113 ferroptosis-related genes in four TNBC cohorts. **(I)** Differences in TRG score between different clinical trait groups in the TCGA cohort.

In contrast, glycolysis, the NOTCH signaling pathway, and protein secretion were positively correlated with the TRG score (Figure 6A). ssGSEA with curated pathway signatures verified that the TRG score was negatively linked with antigen processing machinery, CD8 T effector, and immune checkpoint and positively associated with WNT target pathways (Figure 6B). We show the genes involved in the above-curated pathway signatures with statistical significance in Figure 6C. Most of the genes involved in immune-related pathways were highly negatively correlated with the TRG score. The stromal and immune scores calculated by ESTIMATE were undoubtedly negatively correlated with TRG score in all TNBC cohorts (Figure 6D). The low TRG score group still had a higher FPI than the high TRG score group, but the mRNAsi and TMB showed no significant differences (Figures 6E–G). Due to differences in FPI between TME subtypes and TRG score groups, correlation analysis was conducted between TRG score and the expression of ferroptosis-related genes. We found that the expression of TNFAIP3, SOCS1, IFNG, ATM, ALOX5, PML, ISCU, and GCH1 was significantly negatively correlated with TRG score in four TNBC cohorts (Figure 6H). The TRG score showed no significant differences between the AJCC_T, AJCC_N, and stage groups, meaning that the TRG score was a novel factor regardless of clinical traits (Figure 6I).

### Role of the tumor microenvironment-related gene score in therapy efficacy

**FIGURE 7:**
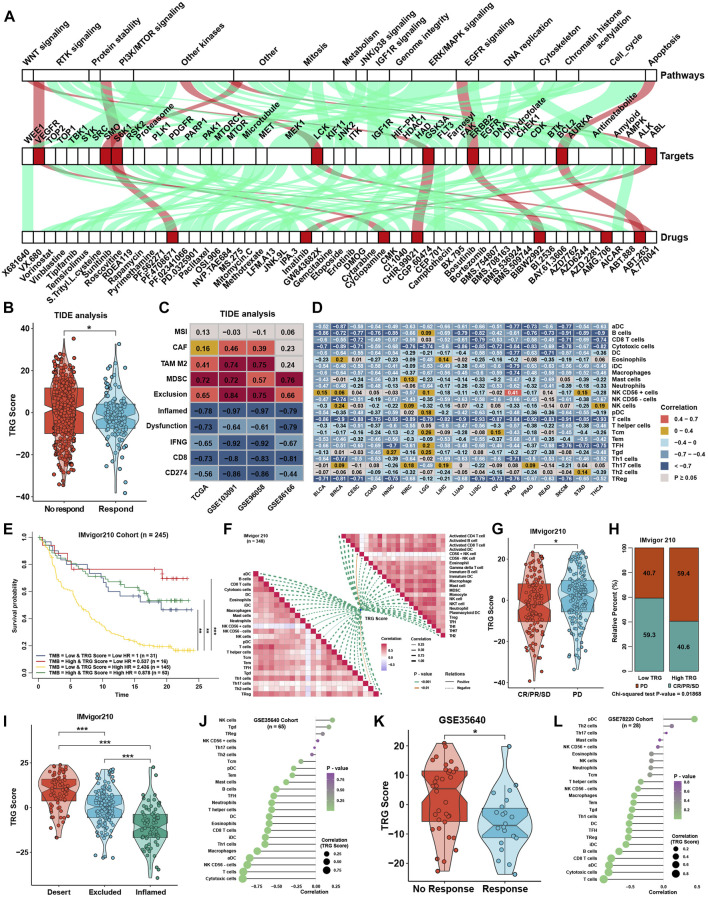
Role of the TRG score in therapeutic efficacy. **(A)** Correlation analysis between TRG score and prediction IC50 values in the whole TNBC cohort. The green line represents that the predicted IC50 of drugs was positively correlated with the TRG score, and the red line represents a negative correlation. **(B)** Difference in TRG score between immunotherapy respondents and nonresponders in TIDE analysis in the whole TNBC cohort. **(C)** Correlation analysis between the TRG score and the results of TIDE analysis in four TNBC cohorts. **(D)** Correlation analysis between the TRG score and the immune cell infiltration levels was calculated with immune cell signature 1 in TCGA pancancer. **(E)** Survival analyses for patients treated with anti-PD-L1 immunotherapy stratified by both TRG score and TMB. **(F)** Correlation analysis between the TRG score and immune cell infiltration levels calculated by immune cell signatures 1 and 2 in the IMvigor210 trial. **(G)** Differences in TRG score between the CR/PR/SD group and the PD group in the IMvigor210 trial. **(H)** Rating clinical response to anti-PD-L1 immunotherapy in high or low TRG score groups in the IMvigor210 cohort using the chi-square test. **(I)** Differences in TRG score among immune phenotypes in the IMvigor210 trial. **(J)** Correlation analysis between the TRG score and the immune cell infiltration levels calculated with immune cell signature 1 in GSE35640. **(K)** The difference in TRG score between immunotherapy respondents and nonresponders in GSE35640. **(L)** Correlation analysis between the TRG score and the immune cell infiltration levels calculated with immune cell signature 1 in GSE78220. The asterisks represent the statistical *p* value (*p < 0.05 and ***p < 0.001).

To explore the association between the TRG score and drug response, we evaluated the estimated IC50 value of 138 drugs included in the GDSC database in four TNBC cohorts. Correlation analyses were conducted between the TRG score and predicted IC50 values (Supplementary Figure S2A). Drugs with significant differences in more than three cohorts were regarded as potential therapeutic drugs; we found that eight drugs were sensitive to the high TRG score group, and 49 drugs were sensitive to the low TRG score group (Figure 7A). The TRG score might logically be related to the efficacy of immunotherapy due to its apparent association with immune cell infiltration and activation. TIDE was utilized to predict the immunotherapy response of TNBC patients, and the TRG score was lower in the immunotherapy response group (Figure 7B). Moreover, TIDE analysis showed that the TRG score was apparently negatively correlated with markers of immunotherapy response and positively correlated with CAFs, myeloid-derived suppressor cells (MDSCs), and TAM M2 (Figure 7C). Lacking TNBC datasets that received immunotherapy, we selected three cohorts that received anti-PDL1, anti-PD1, and anti-MAGE-A3 therapy in bladder cancer (BLCA) and skin melanoma (SKCM) to verify the immunotherapy response prediction value of the TRG score. First, TRG scores were calculated across cancers in TCGA. TRG scores were prognostic risk factors (Supplementary Figure S2B) and were negatively correlated with immune cell infiltration levels in most cancer types, especially in BLCA and SKCM (Figure 7D). Then, we calculated the TRG score in the three immunotherapy cohorts. Interestingly, we found that the TRG score was also a risk factor in IMvigor210 (Supplementary Figure S2C), and patients with a high TRG score and low TMB presented the worst survival advantage (Figure 7E). Correlation analysis further validated that the TRG score was negatively correlated with the expression of MHC, costimulatory, adhesion molecules (Supplementary Figure S2D), and immune cell infiltration levels (Figure 7F). Moreover, a higher TRG score was associated with disease progression (PD), indicating that a higher TRG score might indicate poor response after immunotherapy (Figures 7G,H). As most solid tumors exhibited one of three distinct immunological phenotypes, immune inflamed, immune excluded, or immune desert, studies in the IMvigor210 cohort classified each sample into one of these immune phenotypes (Mariathasan et al., 2018). The immune inflamed phenotype was thought to be rich in immune cell infiltration and sensitive to immunotherapy, while the immune desert was on the contrary. Immune excluded phenotype was surrounded by many immune cells, but the immune cells were confined to the periphery of the tumor cell matrix. We found that a higher TRG score was associated with desert-resistant phenotypes, while inflamed phenotypes possessed a lower TRG score than desert and excluded phenotypes (Figure 7I). In the anti-MAGE-A3 cohort, the TRG score was also negatively correlated with immune cell infiltration levels (Figure 7J) and was lower in the response group (Figure 7K). Similar results could be seen in the anti-PD1 cohort (Figure 7L), although the differences among response groups showed no significance (Supplementary Figure S2E).

### Verification of the tumor microenvironment-related gene score in the external validation TNBC cohort

**FIGURE 8:**
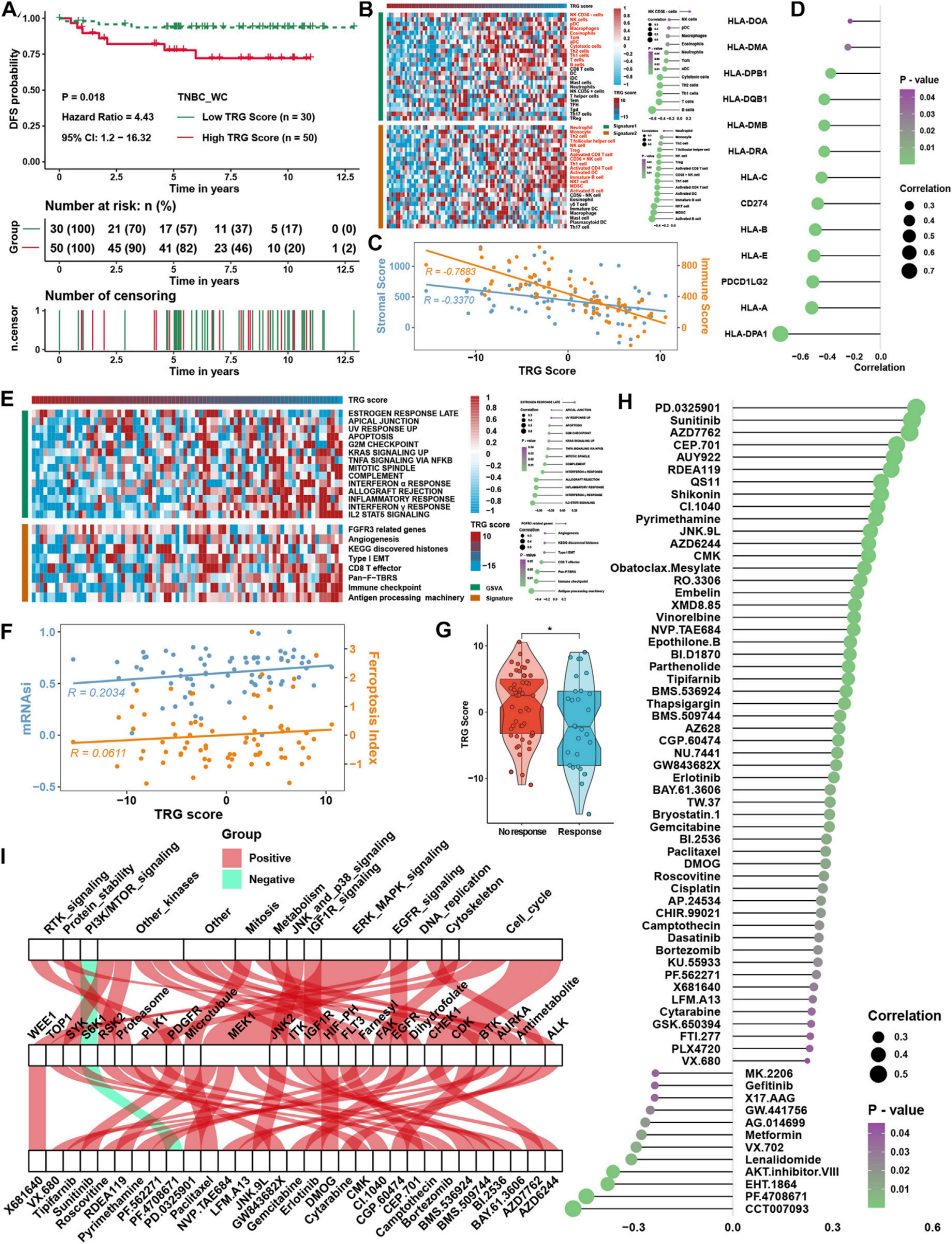
Verification of TRG score in TNBC_WC cohort. **(A)** Survival analysis for the TRG score. **(B)** Heatmap of immune cell infiltration levels calculated with immune cell signatures 1 and 2. **(C)** Correlation analysis between the TRG score and ESTIMATE score, including the stromal score and immune score. **(D)** Correlation analysis between the TRG score and the expression of MHC molecules. **(E)** Correlation analysis between the TRG score and curated pathway enrichment score. **(F)** Correlation analysis between TRG score and FPI and mRNAsi. **(G)** Differences in TRG score between immunotherapy respondents and nonresponders in TIDE analysis. **(H)** Correlation analysis between the TRG score and predicted IC50 values. **(I)** The intersection of GDSC drugs with significance between the results of four TNBC cohorts and the TNBC_WC cohort. The asterisks represent the statistical *p* value (*p < 0.05).

The TRG score was calculated as described earlier in 80 triple-negative breast cancer samples from the West China Hospital (TNBC_WC) cohort. It was found that a higher TRG score was related to the disease progression rate (Figure 8A) and poor survival probability (Supplementary Figure S2F) of TNBC patients. ssGSEA also showed a strong correlation between the TRG score and immune cell infiltration levels in TNBC_WC (Figure 8B), as well as the results of the ESTIMATE score (Figure 8C). Some MHC molecules and ICI targets were also negatively correlated with the TRG score, especially PDL1 and PDCD1LG2 (Figure 8D). For pathway analysis, pathways that were associated with the TRG score in TNBC_WC were almost the same as the results in the four training cohorts (Figure 8E). Correlation analysis showed that the TRG score was positively correlated with the FPI and mRNAsi (Figure 8F). The response group predicted by TIDE analysis showed a lower TRG score than the no response group (Figure 8G). Additionally, we predicted the drug IC50 by GDSC analysis and performed correlation analysis with the TRG score (Figure 8H) and intersection drugs with the results in the training cohorts, as shown in Figure 8I. These results illustrated that the TRG score was a novel and robust method to measure immune cell infiltration levels and therapy efficacy.

### Prognostic signature construction and simplification of the tumor microenvironment-related gene score

**FIGURE 9:**
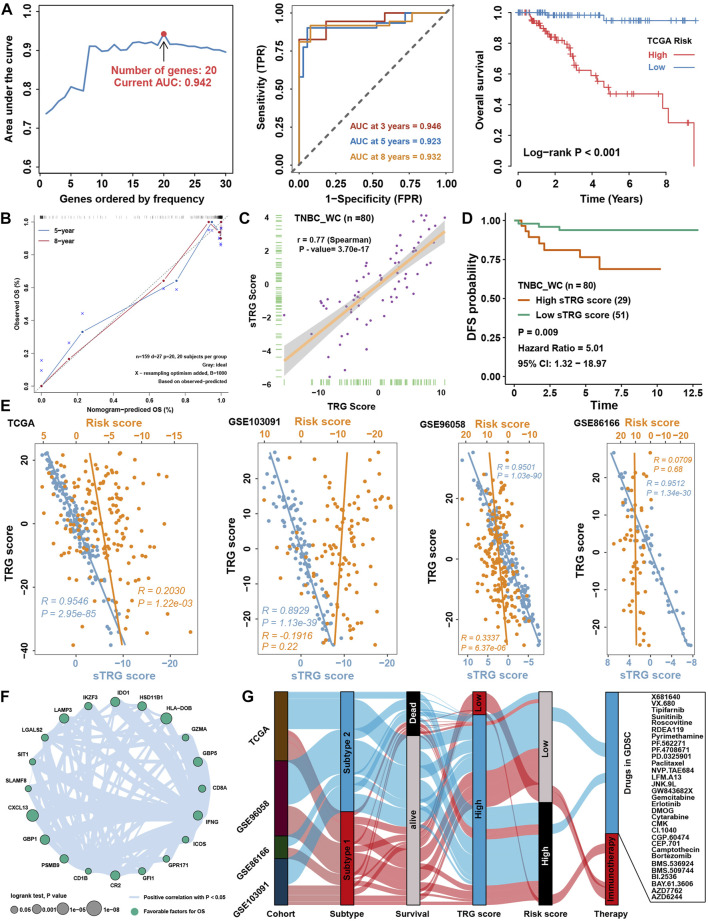
Prognostic signature construction and simplification of the TRG score. **(A)** Iteration LASSO constructed a prognostic signature with 20 genes. The AUC for 5 years was 0.942, and the survival curve for the risk group is shown in the right panel. **(B)** Calibration curve for the comprehensive survival nomogram model in the TCGA TNBC cohort. The dashed diagonal line represents the ideal nomogram, and the blue and red lines represent the 5‐ and 8‐year observed nomograms, respectively. **(C)** Correlation between the sTRG score and TRG score in the TNBC_WC cohort. **(D)** Survival analysis for the sTRG score in the TNBC_WC cohort. **(E)** Correlation analysis between the TRG score and simplified TRG (sTRG) score and risk score in four TNBC cohorts. **(F)** Correlation network among 20 genes involved in the prognostic signature. **(G)** Alluvial diagram shows the changes in relationships among multiple groups in our research.

Considering the accessibility of the TRG score, we aimed to shrink the members of the TRG score and simplify the formula modes to predict the prognosis of TNBC patients. First, survival analysis was processed for 236 TME-related DEGs in TCGA cohorts; 84 genes with a *p* value < 0.05 were selected for further research (Supplementary Figure S3A). Here, iteration LASSO was then used to simplify the members of the TRG score; after multiple attempts to reach the highest 5-year AUC, we finally constructed a prognostic signature with 20 members from TRG score members (Supplementary Table S7; Figure 9A). We could see that there were 20 genes with the most frequencies of occurrence in 1,000 operation iterations in LASSO algorithms, and prognostic signatures with these 20 genes could reach a high area under the curve (AUC) of ROC for 5 years of survival in the TCGA cohort. To provide a convenient approach for predicting the survival probability of a patient with TNBC, we constructed predictive nomograms with the 20 genes generated previously. We developed a nomogram based on the Cox regression model to predict the 5- and 8-year survival probability for TNBC patients (Supplementary Figure S3B). The calibration plots for the 5- and 8-year survival showed an optimal agreement between the nomogram-predicted and observed OS, which was used to evaluate the accuracy of the prediction signature (Figure 9B). For validation of the prognostic value of the 20-gene signature, the patients in the high-risk group showed a worse prognosis than those in the low-risk group, and the same condition could be seen in other TNBC cohorts (Supplementary Figure S3C). Before the prognostic signature was constructed, we conducted a correlation analysis between the TRG score and the expression of 236 TME-related DEGs in four TNBC cohorts. In view of most genes highly correlated with the TRG score, we are supposed to simplify the TRG score by these 20 genes. Surprisingly, the simplified TRG score (sTRG score) calculated based on the expression of these 20 genes was highly positively correlated with the TRG score in the TNBC_WC cohort (Figure 9C), and patients in the high sTRG score group also showed worse DFS than those in the low sTRG score group (Figure 9D). Not unexpectedly, the correlation coefficients between the TRG score and sTRG score were almost close to 1 in other cohorts, which means that they were virtually interchangeable (Figure 9E). However, the risk score showed no significance with the TRG score, indicating that the risk score was a novel factor generated by the iterative LASSO regression model. Eventually, we set up a coexpression network for 20 genes, and we found strong correlations among them (Figure 9F). The visualization of attribute changes in individual patients using an alluvial diagram indicated that the TRG score might be a powerful method to direct therapeutic efficacy or prognostic risk for TNBC patients (Figure 9G).

## Discussion

The evolving immunotherapy of malignant tumors inspired our interest in the role of tumor microenvironment patterns in TNBC. The TME, a critical regulator of disease progression and therapeutic outcome, correlates with patient response to immunotherapy in multiple cancers, with patients possessing immune-favorable TME subtypes benefiting the most from immunotherapy (Bader et al., 2020; Bagaev et al., 2021; Cao et al., 2021). However, previous studies have reported that breast cancer is generally considered a low immune-reactive cancer, TNBC, the most aggressive subtype of breast cancer, but responds to anti-PD-1/PD-L1 immunotherapy (Adams et al., 2019b; Adams et al., 2019c). The urgent question is which types of TNBC patients are suitable for immunotherapy and what their characteristics are. This study was the first to identify TME subtypes of TNBC and TME-related genes represented for each subtype based on consensus clustering analysis. For a more rigorous conclusion, at least two cross-validation methods were chosen for each step, ssGSEA, CIBERSORT, and ESTIMATE, for assessing immune cell infiltration levels and GSEA GSVA and GO analysis for evaluating pathway activation conditions.

Moreover, multiple cohorts were selected for training or validation, and one private external validation cohort was specialized to collect for confirmation of results. All of these innovations will directly differentiate our study of TME patterns in TNBC from all previous studies; there were no systematic studies on the selection of immunotherapy beneficiaries in TNBC based on TME subtypes. The essential light spot of our studies illustrated that two perfect scoring systems based on one gene signature were constructed to predict the efficacy of immunotherapy, response to chemotherapy, and prognosis of TNBC patients.

Previous studies related to the TRG score in TNBC (Qin et al., 2021; Yi et al., 2021; Yang et al., 2022) generally used one method to assess immune cell infiltration levels and did not consider immune-related pathway activation conditions; our study used not only numerous methods to evaluate immune cell infiltration levels but also employed different types of pathway signatures to evaluate pathway enrichment levels. These results provide mechanistic insights into the efficacy of immunotherapy, suggesting that benefits from immunotherapy are not only related to enhanced IL-2/STAT5, IL-6/STAT3, and interferon response pathways but are also associated with inhibition of TGF-β-, NOTCH-, PI3K/AKT-, and EMT-related pathways. Subtype 1 was characterized by multiple infiltrating immune cells, especially cytotoxic cells and antigen-presenting cells, which were reported to correspond to the resistant activated phenotype (Gajewski et al., 2013; Turley et al., 2015; Chen and Mellman, 2017). In contrast, subtype 2 was characterized by a lack of immune cell infiltration, corresponding to the immune-suppressed phenotype, which is often referred to as a “cold” tumor (Kim and Chen, 2016). Most importantly, based on a machine learning method, the PCA score, we successfully converted categorical variables of TME subtypes to numeric variables of the TRG score, which could inherit all characteristics of TME subtypes. The lower the TRG score was, the more likely the patient was to be grouped into subtype 1, indicating better immune cell infiltration levels. The lack of TNBC cohorts that received immunotherapy, TIDE analysis, and immunotherapy cohorts in other tumors was utilized to assess the true power of the TRG score in the prediction of immunotherapy efficacy. We already know that many TNBC patients could benefit from immunotherapy from the Impassion130 study (Schmid et al., 2020; Emens et al., 2021), which was highly consistent with our research. The former critical analysis failed to answer the question of benefit group selection; however, our studies showed that some TNBC patients might have a higher possibility of benefitting from immunotherapy, and the specific cutoff value still needs to be further explored. Although this conclusion failed to be validated in the Impassion130 study due to data permission, we successfully validated the efficacy of the TRG score in other immunotherapy cohorts in metastatic urothelium carcinoma and melanoma treated with anti-PDL1, anti-PD1, and anti-MAGE-A3.

Correlation analysis between TIDE results members and TRG score showed that TRG score was highly positively correlated with CAFs, TAM M2, and MDSC, and all of these cells were reported to be immune suppressive cells and closely associated with cancer stemness (Kwak et al., 2020; Boutilier and Elsawa, 2021; Mao et al., 2021). Subsequent mRNAsi analysis verified these findings that the TRG score was positively correlated with cancer stemness, which might be why subtype 2 was related to progression and metastasis-related pathways and poor immune cell infiltration levels. Cancer stemness has been reported to be associated with immunotherapy efficacy in many studies (Ruiu et al., 2019; Clara et al., 2020; Yang et al., 2020), as well as with drug resistance in TNBC (Cazet et al., 2018; O’Conor et al., 2018). Combined with drug information and sequence data in GDSC, several molecular compounds that might be sensitive in TNBC patients have been identified, broadening the drug research direction of basic experiments in TNBC. Ferroptosis, an iron-dependent form of nonapoptotic cell death that is lethal, has received widespread attention as a potential therapeutic pathway for cancer treatment (Yamaguchi et al., 2013; Ooko et al., 2015). In our research, the ferroptosis level viewed by FPI was higher in subtype 1, meaning that the ferroptosis level might be correlated with an immune-activated TME, and some drugs that infect the ferroptosis process might be sensitive in these patients.

TNBC is a malignant tumor with a poor prognosis, and local recurrence, distant metastasis, and drug-resistant resistance have been the leading cause of death (Bauer et al., 2007; Dent et al., 2007). The constructed TME subtypes and TRG score in this study could reasonably predict the risk of overall survival and had no correlation with previously defined clinical grade and stages, meaning that this score might be a novel factor unaffected by clinical traits. To better predict the survival possibility of TNBC patients, an iterative LASSO algorithm was conducted in TCGA cohorts and validated in three GEO cohorts. Nomograms to indicate survival possibility and death odds were both established by Cox and logistic regression models, which might be helpful in clinical practice. Interestingly, 20 predictive models were accidentally found to construct a simplified TRG score, which might be the same as the TRG score built by 236 DEGs. If the TRG score could be used in clinical practice to predict immunotherapy and chemotherapy efficacy or prognosis of TNBC patients, we suggest that a simplified TRG score might be a more convenient test model.

For 20 genes involved in the predictive signature, we found that these 20 genes were highly associated with immune cell infiltrations in the TME, such as DCs, B cells, and T cells, which were comprehensively reported to be associated with immunotherapy efficacy (Wculek et al., 2020; Sabado et al., 2017; Wang et al., 2019; Guzman-Genuino et al., 2021; Raskov et al., 2021; O'Donnell et al., 2019). LAMP3, IDO1, HSD11B1, and CD1B are markers of DCs; HLA-DOB and CR2 are markers of B cells; and SIT1, IFNG, ICOS, and CXCL13 are markers of T cells. Although some genes were not markers of immune cells, they were reported to be associated with TME and immunotherapy efficacy. The expression of SLAMF8 (Zhang et al., 2021b) and PSMB8 (Kalaora et al., 2020) could predict the efficacy of immune checkpoint inhibitor immunotherapy in gastrointestinal cancer and melanoma. IKZF3 deficiency could potentiate chimeric antigen receptor T cells to target solid tumors (Zou et al., 2022) and activation of the GPR171 pathway could suppress T cell activation and limit antitumor immunity (Fujiwara et al., 2021). Several immune-related molecules, including LGALS2, GFI1, and GBP1/5, have not yet been reported to be related to immunotherapy. Eventually, these results further demonstrated that simplifying the TRG score by 20 immune-related genes was a perfect signature highly correlated with immune cell infiltration levels to predict immunotherapy efficacy.

Although the TRG score could reasonably predict the efficacy of immunotherapy and the prognosis of TNBC patients, to validate all of the abovementioned analyses in public datasets, we finally collected 80 TNBC patients in West China Hospital and performed high-throughput sequencing. The TRG score showed powerful abilities in prognostic prediction and assessing immune cell infiltration levels. Importantly, this cohort was also one of the few sequenced data with clinical information on TNBC; however, patients in this cohort had not yet received immunotherapy. From the TIDE and GDSC analysis results in this cohort, immunotherapy and several drugs identified in the abovementioned research were also validated.

## Conclusion

In conclusion, the TRG score was a convenient method to comprehensively classify the TME subtypes and their corresponding characteristics and pathway activation levels in TNBC. It could also be used to assess some cancer-related features, including the ferroptosis index, genetic variation, drug sensitivity, and mRNAsi of individual patients, and further predict the response to immunotherapy of TNBC patients. Importantly, this study provides a perspective for the comprehensive evaluation of the cellular, molecular, and genetic factors associated with TME infiltration patterns to further reverse TME cell infiltration characterization into “hot tumors”, thus improving the response to an immune checkpoint inhibitor.

## Data Availability

The original contributions presented in the study are included in the article/Supplementary Materials; further inquiries can be directed to the corresponding author.

## Glossary

TNBC: Triple-negative Breast Cancer
ER: Estrogen Receptor
PR: Progesterone Receptor
DFS: Disease-Free Survival
OS: Overall Survival
CAFs: Cancer-associated Fibroblasts
DCs: Dendritic Cells
TAMs: Tumor-associated Macrophages
TME: Tumor Microenvironment
GEO: Gene Expression Omnibus
TCGA: The Cancer Genome Atlas
ssGSEA: Single-sample Gene Set Enrichment Analysis
GSEA: Gene Set Enrichment Analysis
GSVA: Gene Set Variation Analysis
ESTIMATE: Estimation of Stromal and Immune Cells in Malignant Tumors using Expression Data
GO: Gene Ontology
DAVID: Database for Annotation, Visualization and Integrated Discovery
KEGG: Kyoto Encyclopedia of Genes and Genomes
**mRNAsi**: mRNA-based Stemness Index
FPI: Ferroptosis Index
DEGs: Differentially Expressed Genes
PCA: Principal Component Analysis
HR: Hazard Ratios
LASSO: Logistic Least Absolute Shrinkage and Selection Operator
ROC: Receiver Operating Characteristic
AUC: Area Under the Curve
ICI: Immune Checkpoint Inhibitors
TMB: Tumor Mutation Burden
TIDE: Tumor Immune Dysfunction and Exclusion
TRG: TME-related genes
sTRG: Simplified TRG

## References

[B1] Adams S. Gatti-Mays M. E. Kalinsky K. Korde L. A. Sharon E. Amiri-Kordestani L. (2019). Current landscape of immunotherapy in breast cancer: A review. JAMA Oncol. 5 (8), 1205–1214. 10.1001/jamaoncol.2018.7147 30973611 PMC8452050

[B2] Adams S. Loi S. Toppmeyer D. Cescon D. W. De Laurentiis M. Nanda R. (2019). Pembrolizumab monotherapy for previously untreated, PD-L1-positive, metastatic triple-negative breast cancer: cohort B of the phase II KEYNOTE-086 study. Ann. Oncol. 30 (3), 405–411. 10.1093/annonc/mdy518 30475947

[B3] Adams S. Schmid P. Rugo H. S. Winer E. P. Loirat D. Awada A. (2019). Pembrolizumab monotherapy for previously treated metastatic triple-negative breast cancer: cohort A of the phase II KEYNOTE-086 study. Ann. Oncol. 30 (3), 397–404. 10.1093/annonc/mdy517 30475950

[B4] Bader J. E. Voss K. Rathmell J. C. (2020). Targeting metabolism to improve the tumor microenvironment for cancer immunotherapy. Mol. Cell 78 (6), 1019–1033. 10.1016/j.molcel.2020.05.034 32559423 PMC7339967

[B5] Bagaev A. Kotlov N. Nomie K. Svekolkin V. Gafurov A. Isaeva O. (2021). Conserved pan-cancer microenvironment subtypes predict response to immunotherapy. Cancer Cell 39 (6), 845–865.e7. 10.1016/j.ccell.2021.04.014 34019806

[B6] Bao X. Shi R. Zhao T. Wang Y. (2020). Mast cell-based molecular subtypes and signature associated with clinical outcome in early-stage lung adenocarcinoma. Mol. Oncol. 14 (5), 917–932. 10.1002/1878-0261.12670 32175651 PMC7191192

[B7] Bauer K. R. Brown M. Cress R. D. Parise C. A. Caggiano V. (2007). Descriptive analysis of estrogen receptor (ER)-negative, progesterone receptor (PR)-negative, and HER2-negative invasive breast cancer, the so-called triple-negative phenotype: A population-based study from the California cancer registry. Cancer 109 (9), 1721–1728. 10.1002/cncr.22618 17387718

[B8] Bindea G. Mlecnik B. Tosolini M. Kirilovsky A. Waldner M. Obenauf A. C. (2013). Spatiotemporal dynamics of intratumoral immune cells reveal the immune landscape in human cancer. Immunity 39 (4), 782–795. 10.1016/j.immuni.2013.10.003 24138885

[B9] Boutilier A. J. Elsawa S. F. (2021). Macrophage polarization States in the tumor microenvironment. Int. J. Mol. Sci. 22 (13), 6995. 10.3390/ijms22136995 34209703 PMC8268869

[B10] Brueffer C. Vallon-Christersson J. Grabau D. Ehinger A. Hakkinen J. Hegardt C. (2018). Clinical value of RNA sequencing-based classifiers for prediction of the five conventional breast cancer biomarkers: A report from the population-based multicenter Sweden cancerome analysis network-breast initiative. JCO Precis. Oncol. 2, 1–18. 10.1200/PO.17.00135 PMC7446376 32913985

[B11] Cagan R. Meyer P. (2017). Rethinking cancer: Current challenges and opportunities in cancer research. Dis. Model. Mech. 10 (4), 349–352. 10.1242/dmm.030007 28381596 PMC5399574

[B12] Cao R. Yuan L. Ma B. Wang G. Tian Y. (2021). Tumour microenvironment (TME) characterization identified prognosis and immunotherapy response in muscle-invasive bladder cancer (MIBC). Cancer Immunol. Immunother. 70 (1), 1–18. 10.1007/s00262-020-02649-x 32617668 PMC10973753

[B13] Cazet A. S. Hui M. N. Elsworth B. L. Wu S. Z. Roden D. Chan C. L. (2018). Targeting stromal remodeling and cancer stem cell plasticity overcomes chemoresistance in triple negative breast cancer. Nat. Commun. 9 (1), 2897. 10.1038/s41467-018-05220-6 30042390 PMC6057940

[B14] Charoentong P. Finotello F. Angelova M. Mayer C. Efremova M. Rieder D. (2017). Pan-cancer immunogenomic analyses reveal genotype-immunophenotype relationships and predictors of response to checkpoint blockade. Cell Rep. 18 (1), 248–262. 10.1016/j.celrep.2016.12.019 28052254

[B15] Chen D. S. Mellman I. (2017). Elements of cancer immunity and the cancer-immune set point. Nature 541 (7637), 321–330. 10.1038/nature21349 28102259

[B16] Chen H. Yao J. Bao R. Dong Y. Zhang T. Du Y. (2021). Cross-talk of four types of RNA modification writers defines tumor microenvironment and pharmacogenomic landscape in colorectal cancer. Mol. Cancer 20 (1), 29. 10.1186/s12943-021-01322-w 33557837 PMC7869236

[B17] Clara J. A. Monge C. Yang Y. Takebe N. (2020). Targeting signalling pathways and the immune microenvironment of cancer stem cells - a clinical update. Nat. Rev. Clin. Oncol. 17 (4), 204–232. 10.1038/s41571-019-0293-2 31792354

[B18] Cortes J. Cescon D. W. Rugo H. S. Nowecki Z. Im S. A. Yusof M. M. (2020). Pembrolizumab plus chemotherapy versus placebo plus chemotherapy for previously untreated locally recurrent inoperable or metastatic triple-negative breast cancer (KEYNOTE-355): A randomised, placebo-controlled, double-blind, phase 3 clinical trial. Lancet 396 (10265), 1817–1828. 10.1016/S0140-6736(20)32531-9 33278935

[B19] Denkert C. Liedtke C. Tutt A. von Minckwitz G. (2017). Molecular alterations in triple-negative breast cancer-the road to new treatment strategies. Lancet 389 (10087), 2430–2442. 10.1016/S0140-6736(16)32454-0 27939063

[B20] Denkert C. von Minckwitz G. Darb-Esfahani S. Lederer B. Heppner B. I. Weber K. E. (2018). Tumour-infiltrating lymphocytes and prognosis in different subtypes of breast cancer: A pooled analysis of 3771 patients treated with neoadjuvant therapy. Lancet. Oncol. 19 (1), 40–50. 10.1016/S1470-2045(17)30904-X 29233559

[B21] DennisG.Jr.ShermanB. T.HosackD. A.YangJ.GaoW.LaneH. C. (2003). David: Database for annotation, visualization, and integrated discovery. Genome Biol. 4 (5), P3. 10.1186/gb-2003-4-5-p3 12734009

[B22] Dent R. Trudeau M. Pritchard K. I. Hanna W. M. Kahn H. K. Sawka C. A. (2007). Triple-negative breast cancer: Clinical features and patterns of recurrence. Clin. Cancer Res. 13 (15), 4429–4434. 10.1158/1078-0432.CCR-06-3045 17671126

[B23] Emens L. A. Adams S. Barrios C. H. Dieras V. Iwata H. Loi S. (2021). First-line atezolizumab plus nab-paclitaxel for unresectable, locally advanced, or metastatic triple-negative breast cancer: IMpassion130 final overall survival analysis. Ann. Oncol. 32 (8), 983–993. 10.1016/j.annonc.2021.05.355 34272041

[B24] Fujiwara Y. Torphy R. J. Sun Y. Miller E. N. Ho F. Borcherding N. (2021). The GPR171 pathway suppresses T cell activation and limits antitumor immunity. Nat. Commun. 12 (1), 5857. 10.1038/s41467-021-26135-9 34615877 PMC8494883

[B25] Gajewski T. F. Woo S. R. Zha Y. Spaapen R. Zheng Y. Corrales L. (2013). Cancer immunotherapy strategies based on overcoming barriers within the tumor microenvironment. Curr. Opin. Immunol. 25 (2), 268–276. 10.1016/j.coi.2013.02.009 23579075

[B26] Gao G. Wang Z. Qu X. Zhang Z. (2020). Prognostic value of tumor-infiltrating lymphocytes in patients with triple-negative breast cancer: A systematic review and meta-analysis. BMC Cancer 20 (1), 179. 10.1186/s12885-020-6668-z 32131780 PMC7057662

[B27] Geeleher P. Cox N. J. Huang R. S. (2014). Clinical drug response can be predicted using baseline gene expression levels and *in vitro* drug sensitivity in cell lines. Genome Biol. 15 (3), R47. 10.1186/gb-2014-15-3-r47 24580837 PMC4054092

[B28] Goldman M. J. Craft B. Hastie M. Repecka K. McDade F. Kamath A. (2020). Visualizing and interpreting cancer genomics data via the Xena platform. Nat. Biotechnol. 38 (6), 675–678. 10.1038/s41587-020-0546-8 32444850 PMC7386072

[B29] Guzman-Genuino R. M. Hayball J. D. Diener K. R. (2021). Regulatory B cells: Dark horse in pregnancy immunotherapy? J. Mol. Biol. 433 (1), 166596. 10.1016/j.jmb.2020.07.008 32693108

[B30] Hanzelmann S. Castelo R. Guinney J. (2013). Gsva: Gene set variation analysis for microarray and RNA-seq data. BMC Bioinforma. 14, 7. 10.1186/1471-2105-14-7 PMC3618321 23323831

[B31] Hugo W. Zaretsky J. M. Sun L. Song C. Moreno B. H. Hu-Lieskovan S. (2016). Genomic and transcriptomic features of response to anti-PD-1 therapy in metastatic melanoma. Cell 165 (1), 35–44. 10.1016/j.cell.2016.02.065 26997480 PMC4808437

[B32] Jang N. Kwon H. J. Park M. H. Kang S. H. Bae Y. K. (2018). Prognostic value of tumor-infiltrating lymphocyte density assessed using a standardized method based on molecular subtypes and adjuvant chemotherapy in invasive breast cancer. Ann. Surg. Oncol. 25 (4), 937–946. 10.1245/s10434-017-6332-2 29330719

[B33] Jezequel P. Loussouarn D. Guerin-Charbonnel C. Campion L. Vanier A. Gouraud W. (2015). Gene-expression molecular subtyping of triple-negative breast cancer tumours: Importance of immune response. Breast Cancer Res. 17, 43. 10.1186/s13058-015-0550-y 25887482 PMC4389408

[B34] Jiang P. Gu S. Pan D. Fu J. Sahu A. Hu X. (2018). Signatures of T cell dysfunction and exclusion predict cancer immunotherapy response. Nat. Med. 24 (10), 1550–1558. 10.1038/s41591-018-0136-1 30127393 PMC6487502

[B35] Kalaora S. Lee J. S. Barnea E. Levy R. Greenberg P. Alon M. (2020). Immunoproteasome expression is associated with better prognosis and response to checkpoint therapies in melanoma. Nat. Commun. 11 (1), 896. 10.1038/s41467-020-14639-9 32060274 PMC7021791

[B36] Kalluri R. (2016). The biology and function of fibroblasts in cancer. Nat. Rev. Cancer 16 (9), 582–598. 10.1038/nrc.2016.73 27550820

[B37] Kim J. M. Chen D. S. (2016). Immune escape to PD-L1/PD-1 blockade: Seven steps to success (or failure). Ann. Oncol. 27 (8), 1492–1504. 10.1093/annonc/mdw217 27207108

[B38] Kwak T. Wang F. Deng H. Condamine T. Kumar V. Perego M. (2020). Distinct populations of immune-suppressive macrophages differentiate from monocytic myeloid-derived suppressor cells in cancer. Cell Rep. 33 (13), 108571. 10.1016/j.celrep.2020.108571 33378668 PMC7809772

[B39] Lee K. Hwang H. Nam K. T. (2014). Immune response and the tumor microenvironment: How they communicate to regulate gastric cancer. Gut Liver 8 (2), 131–139. 10.5009/gnl.2014.8.2.131 24672653 PMC3964262

[B40] Li Y. Y. Chung G. T. Lui V. W. To K. F. Ma B. B. Chow C. (2017). Exome and genome sequencing of nasopharynx cancer identifies NF-κB pathway activating mutations. Nat. Commun. 8, 14121. 10.1038/ncomms14121 28098136 PMC5253631

[B41] Lin N. U. Vanderplas A. Hughes M. E. Theriault R. L. Edge S. B. Wong Y. N. (2012). Clinicopathologic features, patterns of recurrence, and survival among women with triple-negative breast cancer in the National Comprehensive Cancer Network. Cancer 118 (22), 5463–5472. 10.1002/cncr.27581 22544643 PMC3611659

[B42] Liu Z. He J. Han J. Yang J. Liao W. Chen N. (2021). m6A regulators mediated methylation modification patterns and tumor microenvironment infiltration characterization in nasopharyngeal carcinoma. Front. Immunol. 12, 762243. 10.3389/fimmu.2021.762243 35069534 PMC8776994

[B43] Liu Z. Zhao Q. Zuo Z. X. Yuan S. Q. Yu K. Zhang Q. (2020). Systematic analysis of the aberrances and functional implications of ferroptosis in cancer. iScience 23 (7), 101302. 10.1016/j.isci.2020.101302 32629423 PMC7334617

[B44] Loi S. Drubay D. Adams S. Pruneri G. Francis P. A. Lacroix-Triki M. (2019). Tumor-infiltrating lymphocytes and prognosis: A pooled individual patient Analysis of early-stage triple-negative breast cancers. J. Clin. Oncol. 37 (7), 559–569. 10.1200/JCO.18.01010 30650045 PMC7010425

[B45] Malta T. M. Sokolov A. Gentles A. J. Burzykowski T. Poisson L. Weinstein J. N. (2018). Machine learning identifies stemness features associated with oncogenic dedifferentiation. Cell 173 (2), 338–354. 10.1016/j.cell.2018.03.034 29625051 PMC5902191

[B46] Mantovani A. Marchesi F. Malesci A. Laghi L. Allavena P. (2017). Tumour-associated macrophages as treatment targets in oncology. Nat. Rev. Clin. Oncol. 14 (7), 399–416. 10.1038/nrclinonc.2016.217 28117416 PMC5480600

[B47] Mao X. Xu J. Wang W. Liang C. Hua J. Liu J. (2021). Crosstalk between cancer-associated fibroblasts and immune cells in the tumor microenvironment: New findings and future perspectives. Mol. Cancer 20 (1), 131. 10.1186/s12943-021-01428-1 34635121 PMC8504100

[B48] Mariathasan S. Turley S. J. Nickles D. Castiglioni A. Yuen K. Wang Y. (2018). TGFβ attenuates tumour response to PD-L1 blockade by contributing to exclusion of T cells. Nature 554 (7693), 544–548. 10.1038/nature25501 29443960 PMC6028240

[B49] Marra A. Viale G. Curigliano G. (2019). Recent advances in triple negative breast cancer: The immunotherapy era. BMC Med. 17 (1), 90. 10.1186/s12916-019-1326-5 31068190 PMC6507064

[B50] Mejia-Pedroza R. A. Espinal-Enriquez J. Hernandez-Lemus E. (2018). Pathway-based drug repositioning for breast cancer molecular subtypes. Front. Pharmacol. 9, 905. 10.3389/fphar.2018.00905 30158869 PMC6104565

[B51] Michel L. L. von Au A. Mavratzas A. Smetanay K. Schutz F. Schneeweiss A. (2020). Immune checkpoint blockade in patients with triple-negative breast cancer. Target. Oncol. 15 (4), 415–428. 10.1007/s11523-020-00730-0 32514907

[B52] Mittendorf E. A. Philips A. V. Meric-Bernstam F. Qiao N. Wu Y. Harrington S. (2014). PD-L1 expression in triple-negative breast cancer. Cancer Immunol. Res. 2 (4), 361–370. 10.1158/2326-6066.CIR-13-0127 24764583 PMC4000553

[B53] Mootha V. K. Lindgren C. M. Eriksson K. F. Subramanian A. Sihag S. Lehar J. (2003). PGC-1alpha-responsive genes involved in oxidative phosphorylation are coordinately downregulated in human diabetes. Nat. Genet. 34 (3), 267–273. 10.1038/ng1180 12808457

[B54] Newman A. M. Liu C. L. Green M. R. Gentles A. J. Feng W. Xu Y. (2015). Robust enumeration of cell subsets from tissue expression profiles. Nat. Methods 12 (5), 453–457. 10.1038/nmeth.3337 25822800 PMC4739640

[B55] Nishino M. Ramaiya N. H. Hatabu H. Hodi F. S. (2017). Monitoring immune-checkpoint blockade: Response evaluation and biomarker development. Nat. Rev. Clin. Oncol. 14 (11), 655–668. 10.1038/nrclinonc.2017.88 28653677 PMC5650537

[B56] O'Conor C. J. Chen T. Gonzalez I. Cao D. Peng Y. (2018). Cancer stem cells in triple-negative breast cancer: A potential target and prognostic marker. Biomark. Med. 12 (7), 813–820. 10.2217/bmm-2017-0398 29902924

[B57] O'Donnell J. S. Teng M. W. L. Smyth M. J. (2019). Cancer immunoediting and resistance to T cell-based immunotherapy. Nat. Rev. Clin. Oncol. 16 (3), 151–167. 10.1038/s41571-018-0142-8 30523282

[B58] Ooko E. Saeed M. E. Kadioglu O. Sarvi S. Colak M. Elmasaoudi K. (2015). Artemisinin derivatives induce iron-dependent cell death (ferroptosis) in tumor cells. Phytomedicine 22 (11), 1045–1054. 10.1016/j.phymed.2015.08.002 26407947

[B59] Prabhakaran S. Rizk V. T. Ma Z. Cheng C. H. Berglund A. E. Coppola D. (2017). Evaluation of invasive breast cancer samples using a 12-chemokine gene expression score: Correlation with clinical outcomes. Breast Cancer Res. 19 (1), 71. 10.1186/s13058-017-0864-z 28629479 PMC5477261

[B60] Qin Y. Deng J. Zhang L. Yuan J. Yang H. Li Q. (2021). Tumor microenvironment characterization in triple-negative breast cancer identifies prognostic gene signature. Aging (Albany NY) 13 (4), 5485–5505. 10.18632/aging.202478 33536349 PMC7950290

[B61] Quail D. F. Joyce J. A. (2013). Microenvironmental regulation of tumor progression and metastasis. Nat. Med. 19 (11), 1423–1437. 10.1038/nm.3394 24202395 PMC3954707

[B62] Raskov H. Orhan A. Christensen J. P. Gögenur I. (2021). Cytotoxic CD8+ T cells in cancer and cancer immunotherapy. Br. J. Cancer 124 (2), 359–367. 10.1038/s41416-020-01048-4 32929195 PMC7853123

[B63] Robson M. Im S. A. Senkus E. Xu B. Domchek S. M. Masuda N. (2017). Olaparib for metastatic breast cancer in patients with a germline BRCA mutation. N. Engl. J. Med. 377 (6), 523–533. 10.1056/NEJMoa1706450 28578601

[B64] Rosenberg J. E. Hoffman-Censits J. Powles T. van der Heijden M. S. Balar A. V. Necchi A. (2016). Atezolizumab in patients with locally advanced and metastatic urothelial carcinoma who have progressed following treatment with platinum-based chemotherapy: A single-arm, multicentre, phase 2 trial. Lancet 387 (10031), 1909–1920. 10.1016/S0140-6736(16)00561-4 26952546 PMC5480242

[B65] Ruiu R. Tarone L. Rolih V. Barutello G. Bolli E. Riccardo F. (2019). Cancer stem cell immunology and immunotherapy: Harnessing the immune system against cancer's source. Prog. Mol. Biol. Transl. Sci. 164, 119–188. 10.1016/bs.pmbts.2019.03.008 31383404

[B66] Sabado R. L. Balan S. Bhardwaj N. (2017). Dendritic cell-based immunotherapy. Cell Res. 27 (1), 74–95. 10.1038/cr.2016.157 28025976 PMC5223236

[B67] Savas P. Salgado R. Denkert C. Sotiriou C. Darcy P. K. Smyth M. J. (2016). Clinical relevance of host immunity in breast cancer: From TILs to the clinic. Nat. Rev. Clin. Oncol. 13 (4), 228–241. 10.1038/nrclinonc.2015.215 26667975

[B68] Schmid P. Adams S. Rugo H. S. Schneeweiss A. Barrios C. H. Iwata H. (2018). Atezolizumab and nab-paclitaxel in advanced triple-negative breast cancer. N. Engl. J. Med. 379 (22), 2108–2121. 10.1056/NEJMoa1809615 30345906

[B69] Schmid P. Rugo H. S. Adams S. Schneeweiss A. Barrios C. H. Iwata H. (2020). Atezolizumab plus nab-paclitaxel as first-line treatment for unresectable, locally advanced or metastatic triple-negative breast cancer (IMpassion130): Updated efficacy results from a randomised, double-blind, placebo-controlled, phase 3 trial. Lancet. Oncol. 21 (1), 44–59. 10.1016/S1470-2045(19)30689-8 31786121

[B70] Siegel R. L. Miller K. D. Jemal A. (2020). Cancer statistics, 2020. Ca. Cancer J. Clin. 70 (1), 7–30. 10.3322/caac.21590 31912902

[B71] Simon N. Friedman J. Hastie T. Tibshirani R. (2011). Regularization paths for cox's proportional hazards model via coordinate descent. J. Stat. Softw. 39 (5), 1–13. 10.18637/jss.v039.i05 PMC4824408 27065756

[B72] Su S. Chen J. Yao H. Liu J. Yu S. Lao L. (2018). CD10(+)GPR77(+) cancer-associated fibroblasts promote cancer formation and chemoresistance by sustaining cancer stemness. Cell 172 (4), 841–856. 10.1016/j.cell.2018.01.009 29395328

[B73] Sveen A. Ågesen T. H. Nesbakken A. Meling G. I. Rognum T. O. Liestol K. (2012). ColoGuidePro: A prognostic 7-gene expression signature for stage III colorectal cancer patients. Clin. Cancer Res. 18 (21), 6001–6010. 10.1158/1078-0432.CCR-11-3302 22991413

[B74] Telli M. L. Hellyer J. Audeh W. Jensen K. C. Bose S. Timms K. M. (2018). Homologous recombination deficiency (HRD) status predicts response to standard neoadjuvant chemotherapy in patients with triple-negative or BRCA1/2 mutation-associated breast cancer. Breast Cancer Res. Treat. 168 (3), 625–630. 10.1007/s10549-017-4624-7 29275435

[B75] Turley S. J. Cremasco V. Astarita J. L. (2015). Immunological hallmarks of stromal cells in the tumour microenvironment. Nat. Rev. Immunol. 15 (11), 669–682. 10.1038/nri3902 26471778

[B76] Ulloa-Montoya F. Louahed J. Dizier B. Gruselle O. Spiessens B. Lehmann F. F. (2013). Predictive gene signature in MAGE-A3 antigen-specific cancer immunotherapy. J. Clin. Oncol. 31 (19), 2388–2395. 10.1200/JCO.2012.44.3762 23715562

[B77] Wang S. S. Liu W. Ly D. Xu H. Qu L. Zhang L. (2019). Tumor-infiltrating B cells: Their role and application in anti-tumor immunity in lung cancer. Cell. Mol. Immunol. 16 (1), 6–18. 10.1038/s41423-018-0027-x 29628498 PMC6318290

[B78] Wculek S. K. Cueto F. J. Mujal A. M. Melero I. Krummel M. F. Sancho D. (2020). Dendritic cells in cancer immunology and immunotherapy. Nat. Rev. Immunol. 20 (1), 7–24. 10.1038/s41577-019-0210-z 31467405

[B79] Wilkerson M. D. Hayes D. N. (2010). ConsensusClusterPlus: A class discovery tool with confidence assessments and item tracking. Bioinformatics 26 (12), 1572–1573. 10.1093/bioinformatics/btq170 20427518 PMC2881355

[B80] Yamaguchi H. Hsu J. L. Chen C. T. Wang Y. N. Hsu M. C. Chang S. S. (2013). Caspase-independent cell death is involved in the negative effect of EGF receptor inhibitors on cisplatin in non-small cell lung cancer cells. Clin. Cancer Res. 19 (4), 845–854. 10.1158/1078-0432.CCR-12-2621 23344263 PMC3703145

[B81] Yang A. Wu M. Ni M. Zhang L. Li M. Wei P. (2022). A risk scoring system based on tumor microenvironment cells to predict prognosis and immune activity in triple-negative breast cancer. Breast Cancer 29 (3), 468–477. 10.1007/s12282-021-01326-w 35061208 PMC9021102

[B82] Yang L. Shi P. Zhao G. Xu J. Peng W. Zhang J. (2020). Targeting cancer stem cell pathways for cancer therapy. Signal Transduct. Target. Ther. 5 (1), 8. 10.1038/s41392-020-0110-5 32296030 PMC7005297

[B83] Yi J. Zhong W. Wu H. Feng J. Zouxu X. Huang X. (2021). Identification of key genes affecting the tumor microenvironment and prognosis of triple-negative breast cancer. Front. Oncol. 11, 746058. 10.3389/fonc.2021.746058 34745969 PMC8567753

[B84] Yoshihara K. Shahmoradgoli M. Martinez E. Vegesna R. Kim H. Torres-Garcia W. (2013). Inferring tumour purity and stromal and immune cell admixture from expression data. Nat. Commun. 4, 2612. 10.1038/ncomms3612 24113773 PMC3826632

[B85] Zeng D. Li M. Zhou R. Zhang J. Sun H. Shi M. (2019). Tumor microenvironment characterization in gastric cancer identifies prognostic and immunotherapeutically relevant gene signatures. Cancer Immunol. Res. 7 (5), 737–750. 10.1158/2326-6066.CIR-18-0436 30842092

[B86] Zhang Q. Cheng L. Qin Y. Kong L. Shi X. Hu J. (2021). SLAMF8 expression predicts the efficacy of anti-PD1 immunotherapy in gastrointestinal cancers. Clin. Transl. Immunol. 10 (10), e1347. 10.1002/cti2.1347 PMC8546794 34729183

[B87] Zhang S. S. Chen X. Chen T. T. Zhu J. W. Tang B. X. Wang A. K. (2021). GSA-Human: Genome sequence archive for human. Yi Chuan 43 (10), 988–993. 10.16288/j.yczz.21-248 34702711

[B88] Zou Y. Liu B. Li L. Yin Q. Tang J. Jing Z. (2022). IKZF3 deficiency potentiates chimeric antigen receptor T cells targeting solid tumors. Cancer Lett. 524, 121–130. 10.1016/j.canlet.2021.10.016 34687790

