# Supplementary material for: Systematic evaluation of tumor microenvironment and construction of a machine learning model to predict prognosis and immunotherapy efficacy in triple-negative breast cancer based on data mining and sequencing validation
Source: Front Pharmacol. 2022 Sep 26;13:995555. doi: 10.3389/fphar.2022.995555 (PMC9548553; doi:10.3389/fphar.2022.995555)
Supplement: Supplementary file 1 [file Table2.DOCX]

**Figure S1. Consensus clustering of immune cell infiltration levels in four TNBC cohorts.**


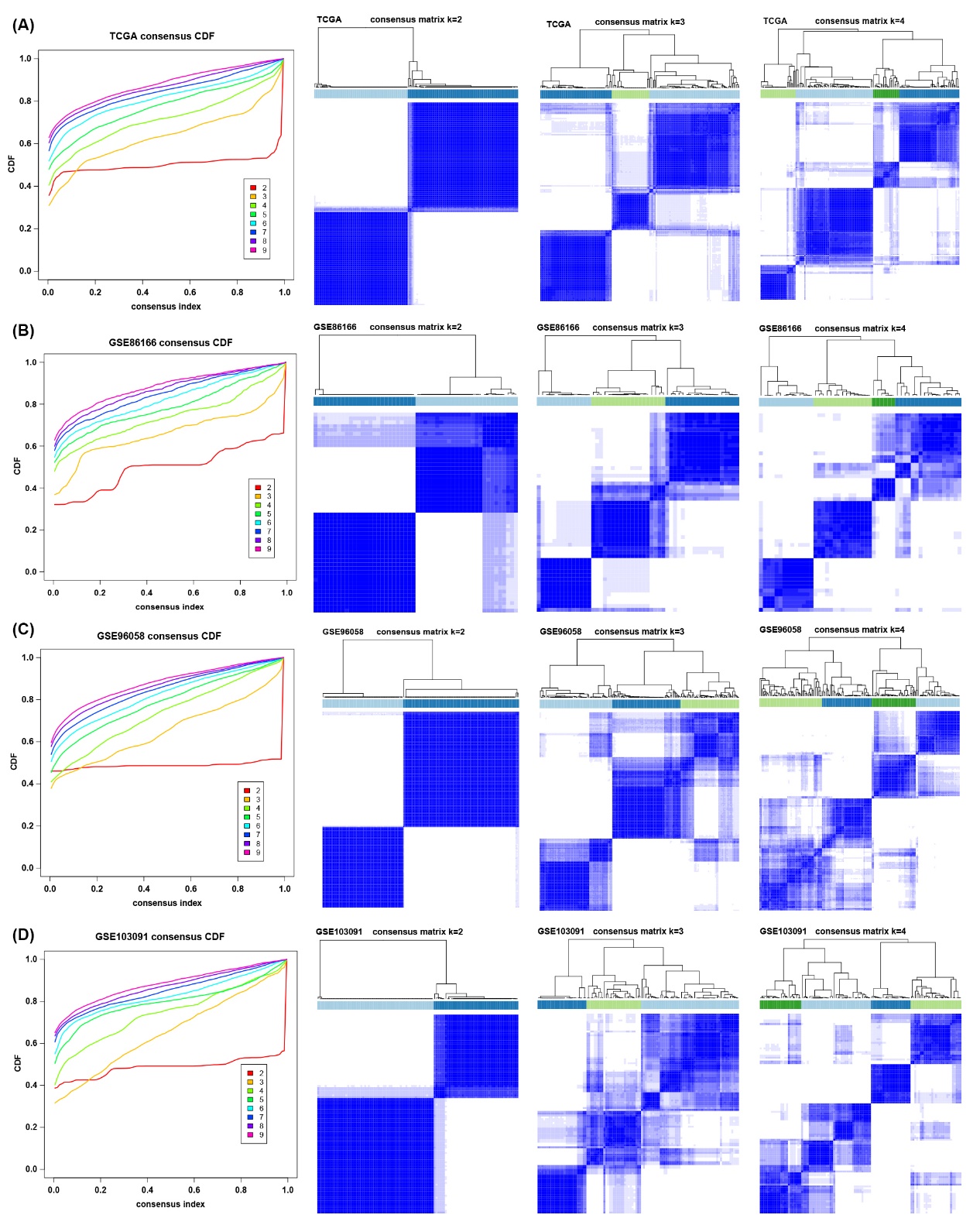


(A-D) Unsupervised clustering of TME in four TNBC cohort and consensus matrices for k = 2 - 4. K = 2 was eventually selected to cluster samples.

**Figure S2. Therapeutic Response Prediction.**


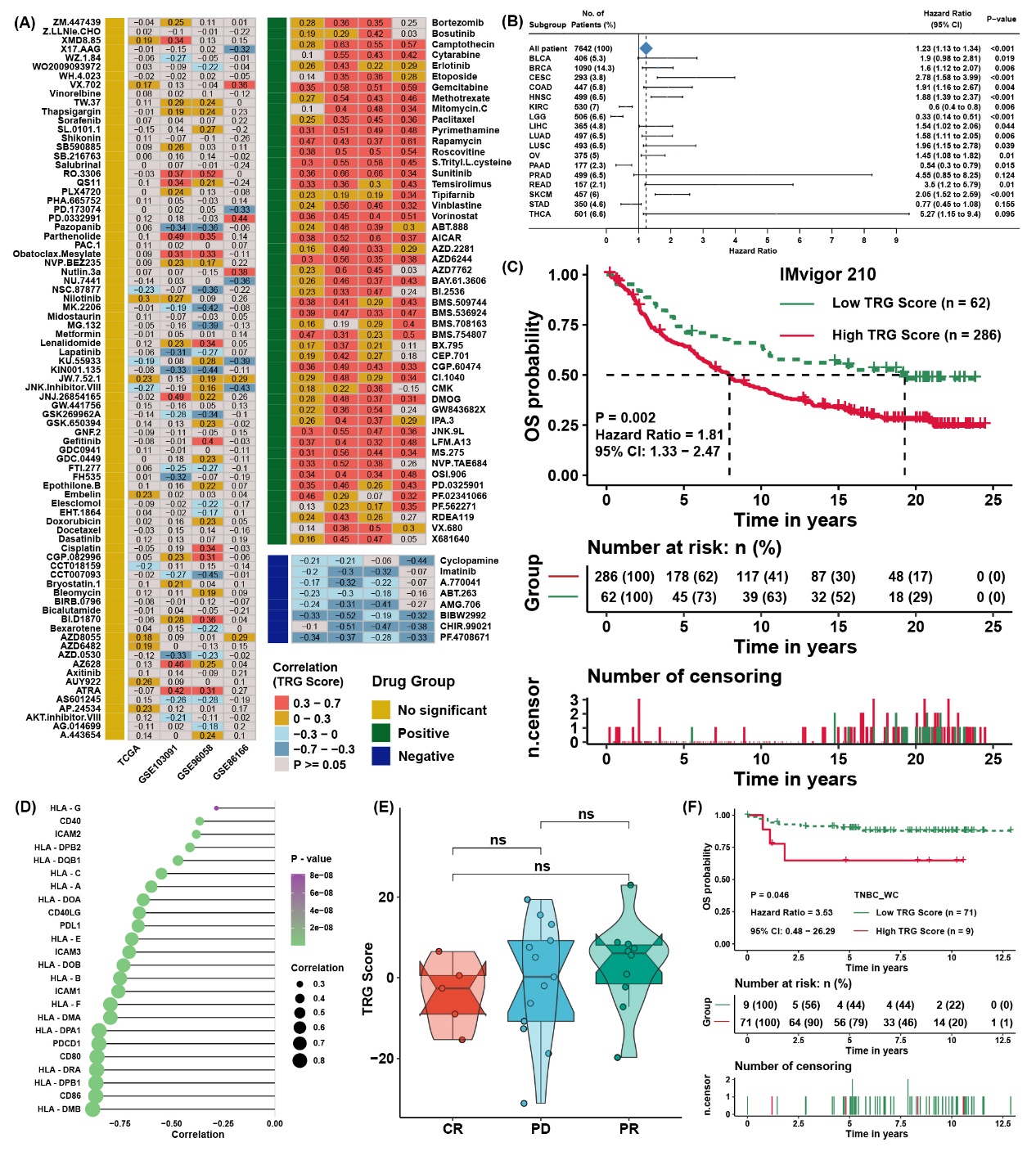


(A) Correlation analysis between prediction IC50 values in GDSC and TRG score in four TNBC cohorts. (B) Forest plot for the TRG score in TCGA pancancer. (C) Survival analysis for the TRG score in the IMvigor210 cohort. (D) Correlation analysis between the TRG score and the expression of MHC molecules in the IMvigor210 cohort. (E) The difference in TRG scores between immunotherapy respondents and nonresponders in GSE78220. (F) Survival analysis for the TRG score in the TNBC_WC cohort.

**Figure S3. Prognostic signature construction and simplification of the TRG score.**

**
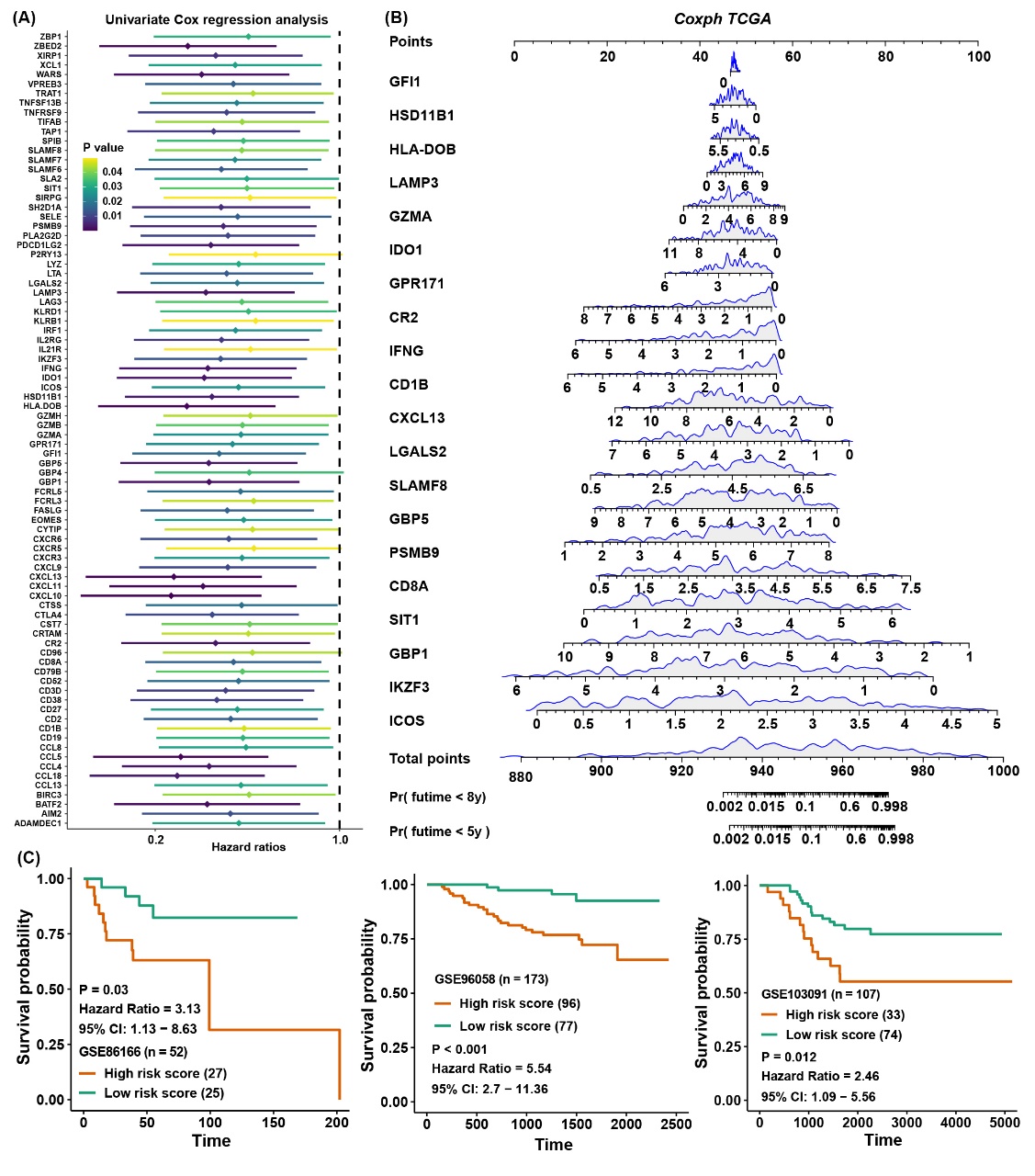
**

(A) Univariate Cox regression analysis of TME-related genes in the TCGA cohort. (B) Nomogram to predict the 5‐y and 8‐y overall survival of TNBC patients. (C) Survival analysis for the risk score in three TNBC cohorts.
